# Effects of individualized blood flow restriction during brief twice-weekly low-load cycling on mobility and muscle function in adults older than 70 years

**DOI:** 10.3389/fspor.2026.1933001

**Published:** 2026-10-02

**Authors:** Alexander Franz, Simon Schroeder, Sanghyeon Ji, Frank Sebastian Fröschen, Ralf-Joachim Schulz, Patrick Wahl

**Affiliations:** 1 Department of Orthopedics and Trauma Surgery, University Hospital Bonn, Bonn, Germany; 2 Department of Trauma and Orthopedic Surgery, BG Klinik Ludwigshafen, Ludwigshafen, Germany; 3 Department of Exercise Physiology, Institute of Exercise Training and Sport Informatics, German Sport University Cologne, Cologne, Germany; 4 The German Research Center of Elite Sport, German Sport University Cologne, Cologne, Germany; 5 Department of Geriatrics, Cellitinnen-Krankenhaus St. Marien, Cologne, Germany

**Keywords:** exercise intervention, functional performance, healthy aging, limb occlusion pressure, muscle strength

## Abstract

**Introduction:**

Age-related declines in muscle strength and physical function increase the risk of mobility limitations, disability, and loss of independence. Although resistance training is effective, many older adults are unable or unwilling to perform high-load exercise, highlighting the need for time-efficient, low-load interventions that preserve physical function. Blood flow restriction (BFR) training may enhance adaptations to low-load exercise, but whether a brief, clinically feasible endurance-based BFR intervention can induce clinically meaningful improvements in mobility remains unclear.

**Methods:**

Twenty adults aged >70 years were randomized to six weeks of supervised low-load cycling (15 min, twice weekly) with either BFR at 60% limb occlusion pressure (INT, *n* = 11) or sham BFR (20mmHg, CON, *n* = 9). Primary outcomes were functional mobility, assessed by the DEMMI, and health-related quality of life. Secondary outcomes included Timed Up and Go (TUG), Six-Minute-Walking-Test (6MWT), lower-limb strength, handgrip strength, body composition, endurance capacity, and cardiovascular safety.

**Results:**

INT showed significantly greater improvements in functional mobility (DEMMI) compared to CON [baseline-adjusted ANCOVA: *p* = 0.02; Hedges’ g [standardized between-group contrast, 95% CI] = 0.91 [−0.04 to 1.87]]. In secondary analyses, no significant between-group differences were observed for TUG [*p* = 0.14; g = −0.68 (−1.61 to 0.25)] or 6MWT distance [*p* = 0.08; g = 0.79 (−0.15 to 1.73)]. However, INT showed significant improvements in lower-limb strength with no relevant improvements in CON [leg press: *p* = 0.04, g = 0.98 [0.02 to 1.94]; leg extension: *p* < 0.01, g = 1.52 [0.49 to 2.56]]. Furthermore, significant improvements were also observed in muscle mass and endurance capacity (*p* < 0.05), as well as in health-related quality of life, including vitality [baseline-adjusted ANCOVA: *p* = 0.042, adjusted mean difference = 6.94 (0.29 to 13.6)] and mental health [*p* < 0.001, g = 1.61 (0.53 to 2.68)]. Handgrip strength was preserved in INT despite a significant decline in CON (*p* = 0.042). In secondary exploratory analyses, HDL cholesterol increased, and HbA1c decreased (*p* < 0.05). Echocardiography revealed no adverse structural or functional cardiac adaptations, and no adverse events occurred.

**Conclusion:**

A brief, twice-weekly low-load cycling program combined with BFR elicited significant rises in lower-limb strength with associated clinically meaningful improvements in mobility despite a minimal exercise dose. Endurance-based BFR appears to be a feasible, safe, and well-tolerated strategy to improve physical function and support independent living in healthy older adults. This trial was not prospectively registered in a public trial registry. It was retrospectively registered at ClinicalTrials.gov (registration number: NCT07825987).

## 1 Introduction

Age-related declines in skeletal muscle mass, strength, and physical function are major contributors to mobility limitations, frailty, loss of independence, and reduced quality of life (QoL) in older adults (1). These progressive impairments are central features of the development of sarcopenia and are associated with an increased risk of falls, hospitalization, institutionalization, and mortality (2), making the preservation of mobility a central objective of healthy ageing and disease prevention (3). Consequently, international guidelines recommend regular resistance and aerobic exercise as key strategies to maintain muscle function and delay the progression towards frailty and sarcopenia (4).

Despite the well-established benefits of exercise, adherence to current physical activity recommendations remains low among older adults (5). Conventional high-load resistance training (HL-RT), although highly effective for improving muscle strength and muscle mass, is frequently limited by musculoskeletal disorders, cardiovascular comorbidities, pain, fear of injury, or the perceived physical demands associated with high mechanical loading (6, 7). Consequently, an important challenge in geriatric exercise medicine is not whether exercise is effective, but whether clinically meaningful improvements in mobility and physical function can be achieved using interventions that are feasible, well tolerated, and require only a minimal exercise dose. Identifying such interventions may improve adherence and facilitate the implementation of exercise into routine preventive care for older adults (8).

Blood-flow restriction (BFR) training has emerged as a promising strategy to augment adaptations to low-load exercise by increasing metabolic stress while minimizing mechanical loading (9). While the efficacy of low-load BFR resistance training is well established (10), considerably less is known about endurance-based BFR protocols in older adults. Existing studies have predominantly investigated walking interventions (11–13), with preliminary supporting evidence also reported in a conference abstract (14), whereas cycling-based BFR protocols remain scarce, despite offering a practical and accessible exercise modality for many older adults in supervised or even unsupervised settings to target muscular, and cardiovascular health simultaneously (15).

However, whether clinically meaningful improvements in mobility and physical function can be achieved by BFR and/or with a minimal effective exercise dose remains largely unknown. Recent systematic reviews concluded that, despite consistent gains in muscle strength and mass, evidence for superior improvements in functional performance and QoL is still inconclusive, largely because of the limited number of well-controlled endurance-based studies (16, 17). However, preserving mobility rather than solely increasing muscle strength is arguably the more relevant clinical objective for maintaining independence in older adults. In this context, the De Morton Mobility Index (DEMMI) represents a validated and responsive assessment of mobility across a broad spectrum of functional abilities and has been shown to detect clinically meaningful changes in geriatric populations (18). Nevertheless, the DEMMI has rarely been incorporated into BFR research, limiting conclusions regarding the clinical relevance of these interventions for maintaining independent living. Since it is known, that through the addition of BFR to an exercise program, time to exhaustion can be significantly reduced by maintaining necessary physiological stimuli for muscular adaptations (19, 20), identification of the lowest exercise dose capable of inducing meaningful functional adaptations would have considerable implications for the implementation of exercise programs in geriatric prevention and rehabilitation.

Additional concerns regarding the safety of BFR training in older adults further contribute to its cautious adoption in clinical and preventive settings. Although accumulating evidence suggests that appropriately prescribed BFR is well tolerated in older adults (21, 22), comprehensive cardiovascular characterization has rarely been included in previous BFR studies. Consequently, the overall clinical profile of individualized endurance-based BFR remains incompletely characterized.

Therefore, the aims of this study were to: 1) determine whether a six-week, twice-weekly, low-load cycling program combined with individualized BFR induces clinically meaningful improvements in functional mobility (DEMMI) and health-related quality of life in community-dwelling adults older than 70 years, compared with identical endurance exercise without effective BFR and 2) characterize the effects of this intervention on muscular strength, body composition, endurance capacity, and cardiovascular safety.

We hypothesized that individualized BFR would enable clinically meaningful improvements in functional mobility and health-related quality of life despite an intentionally minimal exercise dose.

## 2 Material and methods

### 2.1 Participants

Twenty community-dwelling adults (6 men, 14 women; mean ± SD: age 75.7 ± 6.7 years, body mass 61.9 ± 15.5 kg, height 166 ± 7.2 cm) participated in this prospective randomized controlled trial.

Eligibility criteria included an age >70 years, written informed consent, and medical clearance for participation in exercise training with BFR. Exclusion criteria comprised acute or chronic malignancy, immunosuppression, acute or chronic systemic infection, sickle cell anemia, and previous vascular surgery of the lower extremities (e.g., bypass grafting or stent implantation).

Participants were randomly allocated (1:1) using a computer-generated randomization sequence (http://www.randomizer.org) to either:

- **Control group (CON, *n*** **=** **9):** Low-load cycling exercise (LL-END) combined with sham BFR (20 mmHg).
- **Intervention group (INT, *n*** **=** **11):** Identical low-load cycling exercise combined with individualized BFR (60% limb occlusion pressure, LL-BFR-END).

Allocation concealment was achieved using sequentially numbered opaque envelopes prepared by an investigator not involved in recruitment or testing. All participants provided written informed consent before randomization.

The study was approved by the local Ethics Committee of the German Sport University Cologne (Trial-ID: 029/2022; Approval Date: 07.03.2022) and conducted according to the Declaration of Helsinki in its current version (2024). This trial was not prospectively registered; it was retrospectively registered at ClinicalTrials.gov (registration number: NCT07825987).

### 2.2 Study design

This study was designed as a prospective, single-blinded, parallel-group randomized controlled trial investigating whether a brief, clinically feasible, twice-weekly low-load cycling program combined with individualized BFR induces clinically meaningful improvements in functional mobility in healthy adults older than 70 years. The outcome assessor performing all functional, mobility, strength, and exercise testing was blinded to group allocation throughout the study. Given the difference in cuff pressure between INT and CON, participant blinding could not be fully guaranteed and is addressed as a limitation.

This trial is reported in accordance with the CONSORT 2025 guidelines (23). A completed CONSORT checklist mapping each item to the corresponding manuscript section is provided as a Supplementary File.

Baseline assessments were performed within one week before the intervention and distributed over two testing days to minimize fatigue. Day 1 included anthropometry, body composition, blood sampling, ECG and transthoracic echocardiography. Day 2 comprised functional mobility testing [DEMMI, Timed Up and Go (TUG), 6-Minute Walk Test (6MWT)], endurance assessment, and muscle strength testing including familiarization with the one-repetition maximum (1RM) protocol. Post-intervention assessments were conducted 48–72 h after completion of the final training session using the identical testing sequence.

The primary outcomes were functional mobility, assessed by the DEMMI, and health-related quality of life, assessed by the SF-36 domains and summary scores considered jointly as a single outcome family. Secondary outcomes included the TUG, 6MWT, lower-limb and handgrip strength, body composition, endurance performance, and cardiovascular safety. A schematic overview of the study design is provided in Figure 1.

**Figure 1:**
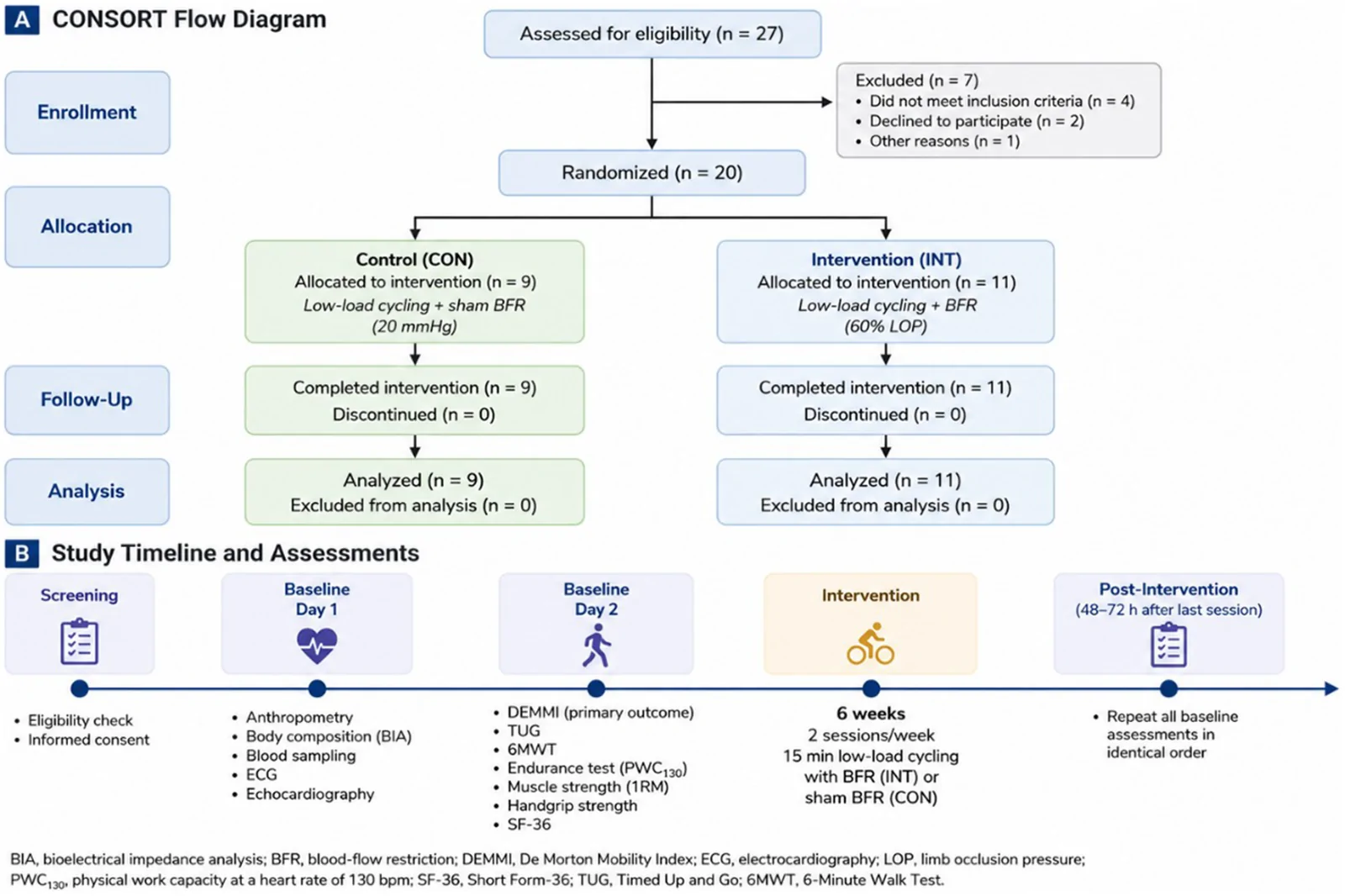
**(A)** CONSORT flow diagram showing participant enrollment, allocation, follow-up, and analysis. Of 27 individuals assessed for eligibility, 7 were excluded (4 did not meet inclusion criteria, 2 declined to participate, 1 for other reasons); 20 participants were randomized to CON (*n* = 9) or INT (*n* = 11); all randomized participants completed the intervention and were included in the analysis, with no discontinuations or exclusions. **(B)** Study timeline and assessment schedule across screening, baseline testing (Day 1 and Day 2), the six-week intervention period, and post-intervention assessment (48–72 h after the final session).

### 2.3 Sample size calculation

The primary outcomes were the DEMMI and health-related quality of life (SF-36). As no pilot data were available on the effects of BFR-augmented endurance training on either outcome at the time of protocol design, the sample size calculation was instead based on published effect sizes for BFR-induced improvements in muscle strength (pooled ES 0.70–3.09) (17, 24, 25). To determine the necessary sample size, we conducted a power analysis using G*Power (version 3.1.9.7). With assumptions of a mean effect size (cohen’s f) of 0.50, an alpha error (ɑ) of 0.05, and statistical power (1 - *β*) of 0.90, the analysis determined a minimum sample size of *N* = 14 (7 per group) for a repeated measures design (within-between interaction, correlation level among repeated measures: 0.50, non-sphericity correction: 1.0). Consequently, the attained sample size of *N* = 20 was considered adequate to evaluate large effects on the muscular strength outcomes, the outcomes on which the power calculation was based. This calculation therefore does not directly power either co-primary outcome. The achieved sample provides only limited precision for the DEMMI and quality-of-life results and should be regarded as exploratory for the secondary functional, strength, and cardiometabolic outcomes.

To assess whether the attained sample size was also adequate for the DEMMI specifically, we performed an additional sensitivity power analysis, estimating the smallest standardized effect our sample of *N* = 20 could detect with power of 0.80. Using the same assumptions as the original calculation, our sample was sufficient to detect a standardized effect of d ≈ 0.66 (a medium-to-large effect) for the DEMMI between-group comparison. Since the actual correlation between participants’ pre- and post-intervention DEMMI scores in our sample (r = 0.78) was higher than the value assumed in the original calculation (r = 0.50), the true sensitivity of our trial was likely even better than this estimate suggests.

### 2.4 Interventions

#### 2.4.1 Limb occlusion pressure & blood flow restriction

To determine the external pressure applied during BFR-Training, the individual limb occlusion pressure (LOP) was measured before every exercise session using a personalized tourniquet system, PTS for BFR, (Delfi Medical Innovations Inc., Vancouver Canada) in combination with Easi-Fit Tourniquet Cuffs (Delfi Medical Innovations Inc., Vancouver, Canada). The BFR device has been validated for measuring an individual’s LOP (26) and incorporates surgical-grade tourniquet autoregulation technology to maintain cuff pressure at a prescribed percentage of LOP throughout exercise (27)*.* The applied tourniquet cuffs were medical-grade, contoured cuffs with 11.5 cm wide, circumferential bladders and were available in multiple lengths (18″, 24″, and 34″). The inflatable tourniquet cuffs were placed proximal on both thighs before the training session, and the LOP was automatically measured by the BFR device after a 10-minute rest period in a supine position in both groups (average LOP across the sessions; INT: 202 mmHg ± 24; CON: 206 mmHg ± 25). Subsequently, participants of the INT received a pressure during the cycling exercise of 60% of the measured LOP (121 ± 14 mmHg), whereas participants of the CON received a fixed sham-pressure of 20 mmHg, which does not meaningfully alter arterial blood flow (28). During LOP and pressure application, participants could not view the device display in either group. All participants were informed that the study compared a higher- and a lower-pressure cuff condition, without being told which condition was expected to restrict blood flow.

### 2.4.2 Cycling exercise

All participants performed six weeks of cycling training, performed twice a week. The exercise sessions were performed in an outpatient setting at the Cellitinnen-Krankenhaus St. Marien, Cologne and were supervised by a sports therapist. The exercise protocol was carried out on a stationary cycling trainer (Thera-trainer tigo, medica Medizintechnik GmbH, Hochdorf, Germany). Exercise intensity was prescribed as an internal training load, individually matched to a target relative heart rate (percentage of heart rate reserve) determined for each participant during baseline testing, rather than to a fixed absolute external workload (29). The intensity of the cycling exercise was individualized as previously described (30). Briefly, the targeted exercise heart rate (EHR) was calculated based on the maximal heart rate (220 – age) and the heart rate reserve [HRR = HR (max) – HR (rest)], while resting heart rate was determined in a sitting position after a 10 min rest period. The corresponding workload in watts was identified during a submaximal incremental cycling ergometer test (start 20W; increase +20W every 3 min). Blood pressure (RR) and HR were measured at each stage, and the test was terminated once the calculated EHR [EHR = HR (rest) + (HRR × 0.5)] was reached.

The training protocol consisted of a 15-minute continuous cycling exercise at the intensity of the corresponding individual EHR. If a participant did not reach the target EHR during a session, workload was increased in the subsequent session. If the target heart rate was exceeded, workload was reduced accordingly (31). No session was terminated due to inability to sustain the prescribed workload. Heart rate during sessions was monitored in real time by the supervising therapist. Training power output, and rating of perceived exertion across the twelve sessions are shown in Supplementary Figure 1. The additional BFR- or sham-protocol was applied bilaterally during the entire cycling sessions.

### 2.5 Outcome measures

The outcome measures were obtained before the start of the six-week intervention period (T0), as well as after the intervention period (48–72 h after the last intervention session, post, T1). All outcome assessments were performed by the same investigator, who was blinded to group allocation and who was not involved in supervising the BFR/sham training sessions.

#### 2.5.1 Primary outcomes: functional mobility & quality of life

Functional mobility was assessed using the De Morton Mobility Index (DEMMI). The cross-culturally adapted German version was used in the present study, which has demonstrated excellent inter-rater reliability (ICC = 0.94) and construct validity in geriatric inpatients (32, 33). Raw item scores were converted to the 0–100 interval-level DEMMI score using the official Rasch-based conversion table published by the instrument developers (34). The assessing investigator completed standardized official DEMMI rater training prior to the study. For the DEMMI, participants performed 15 mobility tasks ranging from independent position changes on a mat or chair to forward and backward walking, tandem stance with eyes closed, and jumping. Each task was scored from 0 to 2, with 2 indicating correct execution and 0 assigned if the task could not be performed. The Rasch converted interval DEMMI score ranges from 0 to 100, with higher scores indicating better mobility. A DEMMI score of 20–36, 39–67, and 74–100 signifies severe, mild to moderate, and no mobility difficulty, respectively (35).

Health-related QoL was assessed using the German cross-culturally adapted and validated version of the Short Form-36 Health Survey (SF-36) (36), based on the original instrument developed by Ware and Sherbourne (37) and further validated in an elderly population (38). The questionnaire was administered under license from the copyright holder and was completed by participants via self-administration. Domain and summary scores were derived using the standard SF-36 scoring algorithm (39). For the purposes of the co-primary outcome, health-related quality of life was operationally defined as the eight SF-36 domains and component summary scores analyzed jointly as a single outcome family, rather than as individually pre-specified endpoints.

Secondary Outcomes

#### 2.5.2 Timed up and go test

The Timed Up and Go (TUG) test is a valid measure for assessing dynamic balance and fall risk in older adults (40). The test was conducted using a chair without armrests and a marker placed three meters away. Participants were instructed to rise from the chair without using their hands, walk around the marker, and return to a seated position. The completion time (in seconds) was recorded.

#### 2.5.3 Six-minute walking test

Walking capacity was assessed using the 6-Minute Walk Test (6MWT), performed in accordance with the ATS/ERS Technical Standard for field walking tests (41). The test was conducted along a flat, straight, 20-meter indoor corridor marked with turnaround points at each end. Participants walked back and forth along this course as many times as possible within six minutes. Standardized encouragement was provided at each minute using the phrases specified in the ATS/ERS technical standard. A familiarization walk was performed prior to the baseline (T0) assessment to limit a learning effect.

#### 2.5.4 Lower-limb strength

To assess voluntary maximal strength of the lower extremities, a one-repetition maximum (1RM) test was performed in accordance with Barbalho et al. (42). Testing was conducted using exercise machines for the leg press and leg extension (proxomed® functional stem/leg extensor, Alzenau, Germany). The protocol began with a load that allowed the participant to perform a maximum of 10 repetitions. The load was then progressively increased to a level permitting only 6–8 repetitions. This procedure was repeated until a load was reached that could be lifted only once with proper technique, representing the 1RM. For familiarization, the test was conducted on two separate days at baseline.

#### 2.5.5 Body composition & grip strength

Whole-body composition was determined by multi-frequency bioelectrical impedance analysis (BIA) using the seca mBCA 515 (seca GmbH & Co. KG, Hamburg, Germany), an 8-point tetrapolar measurement system with stainless-steel electrodes (three pairs of hand electrodes, two pairs of foot electrodes) applied in a standing position. Measurements were performed under standardized fasting and hydration conditions, following instructions to avoid heavy exercise, food, and fluid intake prior to testing and to void the bladder immediately beforehand. Pre- and post-intervention measurements were performed at the same time of day for each participant. Parameters included body weight, body cell mass (surrogate of skeletal muscle mass, BCM), fat mass, and fat-free mass. BCM represents the metabolically active cellular compartment of the body and is considered a surrogate marker of skeletal muscle quantity and nutritional status, particularly in older adults. Owing to the absence of direct imaging techniques, BCM was used as the primary body composition outcome. Device-specific measurement error for body cell mass was not available from the existing validation literature for the seca mBCA and was not assessed within the present study.

In addition, handgrip strength was measured on the dominant upper extremity using a Baseline Hydraulic Hand Dynamometer (LiTE, Fabrication Enterprises, Inc., White Plains, NY, USA). Testing was performed in accordance with the standardized position recommended by the American Society of Hand Therapists (43): participants were seated with the shoulder adducted and neutrally rotated, the elbow flexed at 90°, the forearm in a neutral position, and the wrist between 0° and 30° dorsiflexion. Three trials were performed per timepoint, with approximately 1–2 min of rest between trials. The mean of the three trials was used for analysis.

#### 2.5.6 Endurance capacity

To assess endurance capacity, a physical work capacity test (PWC130) was performed using an incremental cycling ergometer test (Thera-trainer tigo, medica Medizintechnik GmbH, Hochdorf, Germany), starting at 20 W and increasing by 20 W every three minutes. The test was performed in a seated position, at a cadence of 60–80 rpm. The subject was asked to maintain the exertion until volitional exhaustion.

The test was terminated when the first of the following criteria was met: volitional exhaustion (RPE ≥ 8 on the Borg CR-10 scale), heart rate exceeding 130 bpm, or inability to maintain the prescribed cadence. No fixed maximum test duration was applied. The identical protocol and stopping criteria were used at both the pre- and post-intervention assessments. Furthermore, lactate concentration in capillary blood taken from the participantś earlobe was analysed (BIOSEN S-line, EKF-diagnostic GmbH, Barleben, Germany) before and after each step of the incremental test. As an assessment of aerobic performance, the individual exercise intensity (W) corresponding to a blood lactate concentration of 2 mmol·L⁻¹ (W/2 mmol was determined.

#### 2.5.7 Laboratory measurements

Venous blood samples were taken to determine the following parameters: Blood sugar (mg/dL), glycated haemoglobin (HbA1c, mmol/mol), low-density-lipoprotein (LDL, mg/dL), high-density-lipoprotein (HDL, mg/dL), triglycerides (TG, mg/dL), and C-reactive protein (CRP, mg/L) as a marker of systemic inflammation.

#### 2.5.8 Cardiovascular health

In order to evaluate the effects of the applied interventions on cardiovascular health, electrocardiography (ECG) for detailed cardiac function analysis and transthoracic echocardiography (TTE; Philips EPIQ ultrasound system, Philips, Amsterdam, The Netherlands) to determine ejection fraction, ventricular dimensions, and wall thickness were performed. All echocardiographic images were analyzed by a single investigator, blinded to timepoint and group allocation.

### 2.6 Statistics

Statistical analysis was conducted using R (version 4.4.2). Homoscedasticity and normality of the data were visually evaluated through residual plots and Q-Q plots. To compare the changes in measures over time between groups, linear mixed models (*lme4 package, version 1.1.38*) were used. The model formula was specified as *Value∼Time* *×* *Group + (1 | ID)*, where time (Pre, Post) and group (CON, INT) were included as fixed effects, along with their interaction, and participant identity was included as a random intercept to account for between-subject variability and the correlation between repeated measurements within the same individual. With a single random intercept and two timepoints, this specification implies a compound symmetry covariance structure (equal variance at each timepoint and a constant covariance between them) by construction, rather than a structure that was separately fitted or compared against alternatives.

Significant main effects or interactions for time or group were followed up with multiple pairwise *post-hoc* comparisons using Bonferroni correction (*emmeans package, version 2.0.1*). Missing data were handled via maximum likelihood estimation within the linear mixed models, which uses all available data points without requiring imputation, under the assumption that data were missing at random (44). Linear mixed models are particularly well suited to this setting, as they accommodate a varying number of observations per participant without requiring a complete, balanced dataset. No participant was excluded from analysis due to missing values at a single timepoint. Missing data occurred for W/2 mmol (13 of 20 participants with complete pre-post pairs; 7 patients were excluded because of >2 mmol baseline lactate values; CON: 6 of 9, INT: 7 of 11) and SF-36 (one participant did not complete the questionnaire). As a sensitivity analysis, primary and secondary outcomes were additionally analyzed via ANCOVA (*stats package, version 4.4.2*), with the post-intervention value as the dependent variable and the corresponding baseline (Pre) value and group as covariates. For all model contrasts, standardized (Hedges’ g) and unstandardized (adjusted mean difference in change) effect sizes with 95% confidence intervals were calculated. Hedges’ g was computed from the raw, unadjusted between-group difference in change scores, independent of both the mixed model and the ANCOVA; the adjusted mean difference is the Group coefficient from the ANCOVA model. The confidence interval for Hedges’ g was derived from its large-sample analytic standard error (45), applying a central t-distribution; this differs from the standard error underlying the ANCOVA-based adjusted mean difference, and the two intervals are therefore not directly comparable in precision. Following Cohen’s (46) conventional thresholds, Hedges’ g values were classified as small (g = 0.2), medium (g = 0.5), or large (g ≥ 0.8).

To account for multiple comparisons within outcome families comprising several related measures, time × group interaction *p*-values were corrected separately within each family using the Benjamini-Hochberg procedure (47): across the eight SF-36 domains and summary scores, which together constitute the co-primary quality-of-life outcome, and separately within the secondary laboratory, ECG, echocardiography, body composition, and lower-limb strength and grip/endurance families. The DEMMI, as a co-primary outcome comprising a single composite score, was not subject to within-family correction and was tested at the nominal alpha level. The TUG and 6MWT, as individual secondary outcomes, were likewise each tested at the nominal alpha level. No formal adjustment for multiplicity was applied between the two co-primary outcomes (DEMMI and SF-36-based quality of life), given the limited sample size of this trial. For all results, an alpha level of 0.05 was interpreted as statistically significant. Data are expressed as the mean ± standard deviation.

## 3 Results

Of the 27 individuals assessed for eligibility, seven were excluded (four did not meet the inclusion criteria, two declined to participate, and one for other reasons), yielding a final sample of 20 participants who were randomized to the control (CON, *n* = 9) or intervention (INT, *n* = 11) group. All 20 randomized participants completed the six-week intervention and were included in the final analyses (Figure 1). Baseline demographic and clinical characteristics of the CON and INT groups are presented in Table 1. Groups were well balanced at baseline (*p* ≥ 0.05 for all comparisons), with the exception of the SF-36 Vitality domain (*p* = 0.012) and handgrip strength, which showed a numerically large, though not statistically significant, baseline difference (CON: 31.2 ± 7.85 kg vs. INT: 25.5 ± 5.72 kg; *p* = 0.091).

**Table 1:** Baseline characteristics of the control (CON) and intervention (INT) groups.

Characteristic	CON (*n* = 9)	INT (*n* = 11)	*p*-value
Demographics			
Sex, female/male	6/3	8/3	1.00
Age [years]	75.4 ± 3.6	75.9 ± 4.1	0.790
Height [cm]	167 ± 7.30	165 ± 7.70	0.688
Body weight [kg]	68.0 ± 14.4	70.6 ± 17.0	0.717
BMI [kg/m^2]	23.7 ± 4.70	25.7 ± 6.90	0.480
Functional and balance outcomes			
6MWT [m]	503 ± 90	499 ± 87	0.923
6MWT [km/h]	5.03 ± 0.90	5.04 ± 0.91	0.970
TUG [s]	7.06 ± 1.21	7.34 ± 1.67	0.676
DEMMI [points]	86.2 ± 12.8	85.2 ± 13.4	0.861
Quality of life (SF-36)			
Physical functioning index [point]	86 ± 15	80 ± 17	0.431
Role physical index [point]	100 ± 0	90 ± 21	0.168
Bodily pain index [point]	91 ± 14	85 ± 18	0.413
General health perception [point]	73 ± 9	66 ± 9	0.105
Vitality index [point]	69 ± 5	56 ± 13	**0**.**012**
Social functioning [point]	90 ± 10	79 ± 27	0.236
Role-emotional index [point]	100 ± 0	90 ± 23	0.193
Mental health index [point]	67 ± 7	66 ± 16	0.962
Physical component summary [point]	54 ± 3	51 ± 4.	0.054
Mental component summary [point]	51 ± 2	48 ± 8	0.257
Health change [point]	2 ± 1	3 ± 1	0.238
Cardiometabolic and inflammatory markers			
Triglycerides [mg/dl]	124 ± 46.5	147 ± 51.8	0.337
LDL [mg/dl]	115 ± 44.9	123 ± 45.6	0.711
HDL [mg/dl]	72.8 ± 19.6	65.8 ± 18.3	0.457
Glucose [mg/dl]	123 ± 13.4	117 ± 8.96	0.312
HbA1c [mmol/mol]	41.2 ± 5.1	40.9 ± 6.03	0.896
Cardiologic outcomes			
HR [bpm]	67.6 ± 9.85	71.7 ± 8.55	0.332
RRsys [mmHg]	136 ± 15.4	139 ± 10.6	0.695
RRdia [mmHg]	83.2 ± 6.78	90.3 ± 12.0	0.117
QT [ms]	402 ± 21.1	387 ± 18.9	0.113
QRS [ms]	95.4 ± 11.5	96.7 ± 13.0	0.818
PQ [ms]	183 ± 37.0	181 ± 20.8	0.903
LVOT [cm]	19.9 ± 1.45	19.9 ± 1.92	0.979
LVIDd [cm/m2]	42.2 ± 4.24	40.3 ± 4.73	0.356
LVM [g]	82.2 ± 19.3	69.6 ± 13.8	0.124
Aorta [mm]	32.6 ± 2.51	31.4 ± 2.50	0.304
IVSd [cm]	9.39 ± 2.47	9.42 ± 2.26	0.978
LA-Volume [ml/m2]	31.3 ± 6.95	29.6 ± 6.83	0.618
Ejection fraction [%]	56.1 ± 4.70	57.5 ± 4.59	0.502
Lower-limb strength (1RM)			
Leg press [kg]	85.6 ± 36.3	93.2 ± 25.4	0.603
Leg extension [kg]	36.1 ± 16.5	36.4 ± 14.0	0.963
Grip strength and endurance capacity			
Handgrip strength [kg]	31.2 ± 7.85	25.5 ± 5.72	0.091
Total time, incremental cycling test [min]	13.7 ± 3.04	13.6 ± 3.11	0.983
W/2 mmol [W]	35.3 ± 25.3 (*n* = 6)	56.9 ± 25.3 (*n* = 8)	0.143
Body composition			
Body water [kg]	37.4 ± 7.30	37.0 ± 5.78	0.879
Body fat [kg]	24.4 ± 6.96	26.1 ± 9.51	0.654
BCM [kg]	24.8 ± 7.75	24.0 ± 3.89	0.784
CMF [%]	46.2 ± 6.29	46.5 ± 2.32	0.911
Phase angle [°]	4.80 ± 1.00	4.82 ± 0.33	0.960

Values are mean ± SD unless otherwise indicated. BMI, body mass index; 6MWT, six-minute walking test; DEMMI, De Morton Mobility Index; TUG, timed-up and go test; LDL, low-density lipoprotein; HDL, high-density lipoprotein; HbA1c, glycated haemoglobin; RRsys, systolic blood pressure; RRdia, diastolic blood pressure; LVOT, left ventricular outflow tract diameter; LVIDd, left ventricular internal diameter in diastole; LVM, left ventricular mass; IVSd, interventricular septum diameter in diastole; LA, left atrium; W/2 mmol, exercise intensity corresponding to a blood lactate concentration of 2 mmol·L⁻¹; BCM, body cell mass; CMF, cellular mass fraction. group difference for sex distribution was assessed using Fisher’s exact test. For the SF-36 domains, INT *n* = 10 (one participant did not complete the questionnaire).

### 3.1 Functional mobility

Table 2 summarizes functional and balance outcomes before and after the intervention. A main effect of time was observed for the 6MWT and TUG, indicating improvements from pre to post, irrespective of group (*p* ≤ 0.046). DEMMI showed both a main effect of time and a time × group interaction; *post hoc* tests indicated an increase only in INT (*p* = 0.001), with no change in CON (*p* = 0.593). This was corroborated by a baseline-adjusted ANCOVA [*p* = 0.018, adjusted mean difference = 6.64 points [95% CI: 1.28 to 12.00]; Hedges’ g = 0.91 [95% CI: −0.04 to 1.87]].

**Table 2:** Functional and balance outcomes before (pre) and after (post) the intervention in the control (CON) and intervention (INT) groups.

Outcome	CON (*n* = 9)		INT (*n* = 11)		*p*-values from LMM			*p*-values from ANCOVA	Adj. between-group difference in change [95% CI]	Hedges’ g [95% CI]
	Pre	Post	Pre	Post	Time	Group	Time × Group			
6MWT [m]	503 ± 89.8	506 ± 71.0	499 ± 87.4	543 ± 95.8	**0.037**	0.645	0.068	0.081	40.4 [−5.52, 86.2]	0.79 [−0.15, 1.73]
6MWT [km/h]	5.03 ± 0.90	5.06 ± 0.71	5.04 ± 0.91	5.30 ± 0.91	0.223	0.711	0.337	0.318	0.24 [−0.25, 0.72]	0.40 [−0.51, 1.32]
TUG [s]	7.06 ± 1.21	6.67 ± 1.27	7.34 ± 1.67	6.53 ± 1.24	**<0.001**	0.910	0.113	0.135	−0.36 [−0.84, 0.12]	−0.68 [−1.61, 0.25]
DEMMI [points]	86.2 ± 12.8	87.6 ± 10.4	85.2 ± 13.4	93.5 ± 9.45***	**0.006**	0.604	**0**.**037**	**0**.**018**	6.64 [1.28, 12.0]	0.91 [−0.04, 1.87]

Values are mean ± SD. 6MWT, six-minute walking test; ANCOVA, analysis of covariance; CI, confidence interval; DEMMI, De Morton Mobility Index; LMM, linear mixed model; TUG, timed-up and go test. Significant difference vs. Pre within the group. The adjusted mean between-group difference in change [95% CI] is from a baseline-adjusted ANCOVA, conducted as a sensitivity analysis estimating the same between-group contrast. Hedges’ g [95% CI] is a bias-corrected standardized effect size for the between-group contrast.

* *p* < 0.05

** *p* < 0.01

*** *p* < 0.001

Bold values indicate a statistically significant within-group Pre-to-Post difference (*p* < 0.05).

### 3.2 Quality of life

Assessments for subjective QoL were performed by SF-36. Outcomes are illustrated in Table 3. After Benjamini-Hochberg correction for multiple comparisons, linear mixed-effects models showed significant time × group interactions for General Health Perception, the Physical Component Summary, the Mental Health Index, Reported Health Change, and Vitality, indicating greater improvements in INT compared with CON. Domain scores were derived using the standard SF-36 scoring algorithm. Baseline-adjusted ANCOVA corroborated these effects except for General Health Perception and Social Functioning, which did not remain significant after adjustment for baseline scores (Table 3).

**Table 3:** Health-related quality of life outcomes assessed by short-form 36- score (SF−36) before (pre) and after (post) the intervention in the control (CON) and intervention (INT) groups.

Outcome	CON (*n* = 9)		INT (*n* = 10)		*p*-values from LMM			*p*-values from ANCOVA	Adj. between-group difference in change [95% CI]	Hedges’ g [95% CI]
					Time	Group	Time × Group			
	Pre	Post	Pre	Post						
GH [points]	73 ± 9	74 ± 7	66 ± 9	74 ± 6**	**0**.**009**	0.280	**0**.**044**	0.153	3.70 [−1.53, 8.93]	1.05 [0.06, 2.05]
PCS [points]	54 ± 3*	55 ± 2	51 ± 4	56 ± 2**	**<0**.**001**	0.336	**0**.**005**	**0**.**005**	2.51 [0.89, 4.12]	1.67 [0.58, 2.76]
MH [points]	67 ± 7	71 ± 6**	66 ± 15	79 ± 12**	**<0**.**001**	0.436	**0**.**005**	**<0**.**001**	7.88 [4.57, 11.2]	1.61 [0.53, 2.68]
BP [points]	91 ± 14	96 ± 7	85 ± 17	100 ± 0	**0**.**003**	0.760	0.191	0.051	4.40 [−0.02, 8.81]	0.64 [−0.31, 1.59]
PF [points]	86 ± 15	92 ± 6	80 ± 17	93 ± 12	**<0**.**001**	0.596	0.216	0.320	3.01 [−3.21, 9.24]	0.56 [−0.39, 1.50]
MCS [points]	51 ± 2	52 ± 1	48 ± 8	51 ± 8	**0**.**006**	0.421	0.191	0.244	1.74 [−1.31, 4.78]	0.61 [−0.34, 1.56]
HT [points]	2 ± 1	3 ± 1	3 ± 1	2 ± 1**	0.245	0.769	**0**.**024**	**0**.**035**	−0.84 [−1.62, −0.07]	−1.21 [−2.22, −0.20]
RE [points]	100 ± 0	100 ± 0	90 ± 22	83 ± 36	0.584	0.086	0.584	0.321	−13.0 [−39.9, 13.9]	−0.23 [−1.16, 0.70]
RP [points]	100 ± 0	94 ± 11	90 ± 21	100 ± 0	0.553	0.553	**0**.**068**	0.162	5.56 [−2.47, 13.6]	0.87 [−0.10, 1.84]
SF [points]	90 ± 10	90 ± 10	79 ± 27	89 ± 15**	**0**.**038**	0.378	**0**.**068**	0.063	5.07 [−0.30, 10.4]	0.93 [−0.05, 1.90]
VT [points]	69 ± 5*	74 ± 5**	56 ± 13	71 ± 13**	**<0**.**001**	0.054	**0**.**005**	**0**.**042**	6.94 [0.29, 13.6]	1.56 [0.49, 2.63]

Values are presented as mean ± SD. GH, general health; PCS, physical component summary; MH, mental health; BP, bodily pain; PF, physical functioning; MCS, mental component summary; HT, health transition; RE, role emotional; RP, role physical; SF, social functioning; VT, vitality; ANCOVA, analysis of covariance; CI, confidence interval; LMM, linear mixed model. Time × group interaction *p*-values are adjusted for multiple comparisons within this outcome family using the Benjamini-Hochberg procedure. The adjusted mean between-group difference in change [95% CI] is from a baseline-adjusted ANCOVA, conducted as a sensitivity analysis estimating the same between-group contrast. Hedges’ g [95% CI] is a bias-corrected standardized effect size for the between-group contrast.

* *p* < 0.05 different to INT within the time point.

** *p* < 0.05 different to Pre within the group.

Bold values indicate a statistically significant within-group Pre-to-Post difference (*p* < 0.05).

### 3.3 Lower-limb strength

Lower-limb strength assessed by 1RM, showed a main effect of time for leg press (*p* = 0.003) but not for leg extension (*p* = 0.068). A significant time × group interaction was observed for both exercises (leg press: *p* = 0.026, q = 0.026; leg extension: *p* = 0.001, q = 0.003), whereas no main effect of group was detected (*p* ≥ 0.239). *Post-hoc* analysis revealed significant pre- to post-intervention increases in 1RM in the INT group for both leg press and leg extension (*p* < 0.001), while no significant changes were observed in the CON group (*p* ≥ 0.05) (Figure 2). Baseline-adjusted ANCOVA yielded consistent effects for both leg press [*p* = 0.039, adjusted mean difference = 17.4 kg [95% CI: 1.01 to 33.8]; Hedges’ g = 0.98 [0.02, 1.94]] and leg extension [*p* = 0.002, adjusted mean difference = 9.23 kg [95% CI: 3.85 to 14.6]; Hedges’ g = 1.52 [0.49, 2.56]].

**Figure 2:**
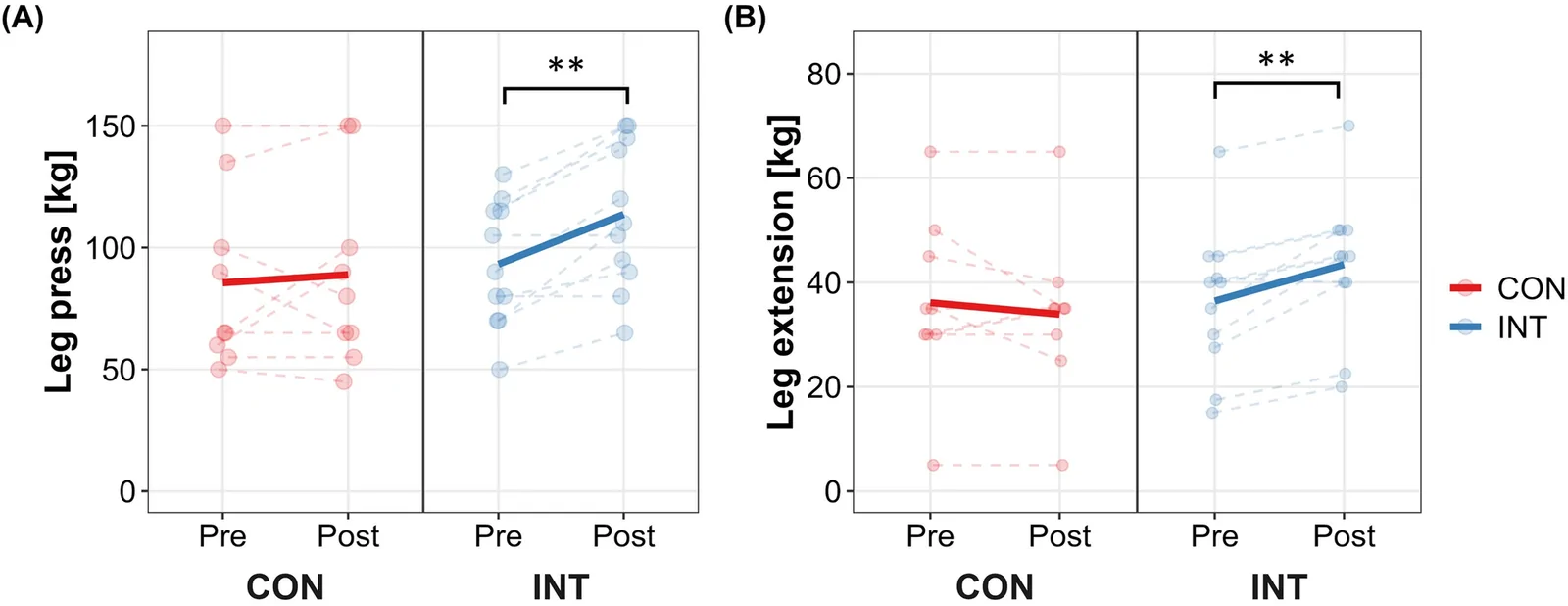
Muscle strength, assessed by one-repetition maximum (1RM) during leg press **(A)** and leg extension **(B)** before (pre) and after (post) the intervention in the control (CON) and intervention (INT) groups. Points represent individual participants; dashed lines indicate within-participant changes; thick lines show group means. The value above each panel is the adjusted mean between-group difference in change and its 95% confidence interval, from a baseline-adjusted analysis of covariance. ***p < 0.001, within-group Pre-to-Post difference (Bonferroni-corrected *post-hoc* comparison).

### 3.4 Grip strength & endurance capacity

For handgrip strength, a significant time × group interaction was found (*p* = 0.007, q = 0.022), without significant main effects of time (*p* = 0.718) or group (*p* = 0.300). *Post-hoc* tests showed a significant decrease in CON from 31.2 ± 7.85 kg to 28.7 ± 10.1 kg (*p* = 0.042), whereas no significant change was observed in INT group (Pre: 25.5 ± 5.72 kg vs. Post: 27.5 ± 7.02 kg, *p* = 0.076). This effect remained significant after adjusting for baseline strength in a sensitivity ANCOVA [*p* = 0.005, adjusted mean difference = 5.46 kg [95% CI: 1.84 to 9.08]; Hedges’ g = 1.23 [95% CI: 0.23 to 2.22]]. *Post-hoc* inspection of individual trajectories suggested that this decline in CON was not entirely uniform: two of nine participants showed comparatively large decreases (−11 kg and −10 kg, respectively), while the remaining seven changed comparatively little.

The total time during the incremental cycling test (Figure 3) showed a main effect of time (*p* = 0.006) and a time × group interaction (*p* = 0.011, q = 0.022), with no main effect of group (*p* = 0.347). *Post hoc* comparisons indicated that the total time increased in the INT group (*p* < 0.001) but not in the CON group (*p* = 0.867). A baseline-adjusted ANCOVA showed a similar effect [*p* = 0.011, adjusted mean difference = 2.33 min [95% CI: 0.62–4.05]; Hedges’ g = 1.14 [95% CI: 0.16 to 2.12]].

**Figure 3:**
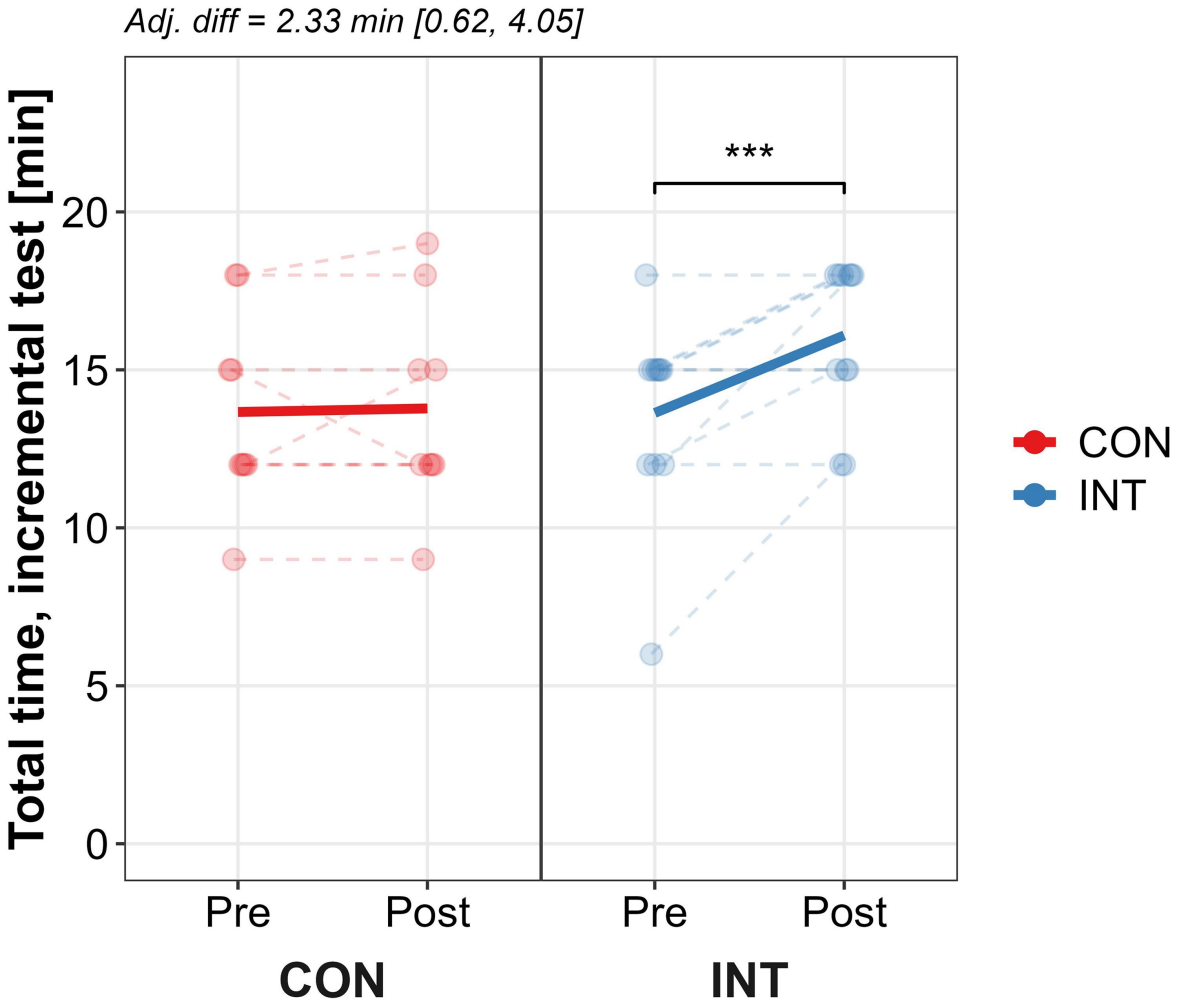
Total time during the incremental cycling test before (pre) and after (post) the intervention in the control (CON) and intervention (INT) groups. Points represent individual participants; dashed lines indicate within-participant changes; thick lines show group means. The value above the panel is the adjusted mean between-group difference in change and its 95% confidence interval, from a baseline-adjusted. ***p < 0.001, within-group Pre-to-Post difference (Bonferroni-corrected *post-hoc* comparison).

The W/2 mmol indicated a significant time effect (*p* = 0.050), with no group (*p* = 0.235) and time × group interaction effect (*p* = 0.223, q = 0.223). *Post hoc* comparisons did not detect significant pre-post differences (*p* = 0.066; CON, Pre: 35.3 ± 25.3 W (*n* = 6), Post: 51.1 ± 32.6 W (*n* = 7); INT, Pre: 56.9 ± 25.3 W (*n* = 8), Post: 61.6 ± 21.7 W (*n* = 7)). Also, a baseline-adjusted ANCOVA showed no significant between-group difference [*p* = 0.452, adjusted mean difference = −8.25 W [95% CI: −31.7 to 15.2]; Hedges’ g = −0.59 [95% CI: −1.76 to 0.57]].

### 3.5 Body composition

Body water (CON, Pre: 37.4 ± 7.30 kg, Post: 38.3 ± 7.90 kg; INT, Pre: 37.0 ± 5.78 kg, Post: 38.1 ± 6.74 kg) and body fat (CON, Pre: 24.4 ± 6.96 kg, Post: 23.7 ± 6.63 kg; INT, Pre: 26.1 ± 9.51 kg, Post: 23.6 ± 9.96 kg) showed a significant effect of time (*p* = 0.008), with body water increasing and body fat decreasing from pre to post (*p* ≤ 0.011). Baseline-adjusted ANCOVA showed no significant between-group difference for either body water [*p* = 0.615, adjusted mean difference = 0.35 kg [95% CI: −1.08 to 1.77]; Hedges’ g = 0.18 [95% CI: −0.73 to 1.09]] or body fat [*p* = 0.139, adjusted mean difference = −1.74 kg [95% CI: −4.10 to 0.63]; Hedges’ g = −0.70 [95% CI: −1.64 to 0.23]].

Body cell mass (BCM) and cellular mass fraction (CMF) showed main effects of time (*p* ≤ 0.036) and time × group interactions (BCM: *p* = 0.001, q = 0.007; CMF: *p* = 0.004, q = 0.011), with no main effect of group (*p* ≥ 0.713). *Post hoc* comparisons indicated increases in INT [BCM: 24.0 ± 3.89 kg vs. 24.9 ± 3.83 kg, an absolute change of 0.94 ± 0.84 kg (+4.1 ± 3.6%); CMF: 46.5 ± 2.32% vs. 47.2 ± 1.92%; both *p* < 0.001], but not in CON [BCM: 24.8 ± 7.75 kg vs. 24.6 ± 7.52 kg, an absolute change of −0.18 ± 0.45 kg (−0.6 ± 1.5%); CMF: 46.2 ± 6.29% vs. 46.1 ± 6.30%; *p* ≥ 0.448]. These interaction effects were corroborated by baseline-adjusted ANCOVA for both BCM [*p* = 0.002, adjusted mean difference = 1.09 kg [95% CI: 0.44 to 1.74]; Hedges’ g = 1.55 [95% CI: 0.51 to 2.59]] and CMF [*p* = 0.006, adjusted mean difference = 0.93% [95% CI: 0.30 to 1.55]; Hedges’ g = 1.34 [95% CI: 0.33 to 2.35]].

Phase angle showed a time × group interaction (*p* = 0.025, q = 0.050) without main effects of time (*p* = 0.124) or group (*p* = 0.592). Phase angle increased in INT (4.82 ± 0.33° vs. 5.04 ± 0.39°, *p* = 0.009) but not in CON (4.80 ± 1.00° vs. 4.76 ± 0.78°, *p* = 0.604). This was similarly reflected in a baseline-adjusted ANCOVA [*p* = 0.012, adjusted mean difference = 0.27° [95% CI: 0.07 to 0.47]; Hedges’ g = 0.99 [95% CI: 0.03 to 1.95]].

ECM (CON, Pre: 27.6 ± 2.26 kg, Post: 27.6 ± 2.98 kg; INT, Pre: 27.9 ± 4.13 kg, Post: 28.1 ± 3.84 kg) showed no effects of time (*p* = 0.289), group (*p* = 0.755), or their interaction (*p* = 0.257, q = 0.308). Consistently, a baseline-adjusted ANCOVA found no significant group difference [*p* = 0.296, adjusted mean difference = 0.34 kg [95% CI: −0.33 to 1.00]; Hedges’ g = 0.48 [95% CI: −0.44 to 1.39]].

### 3.6 Laboratory and cardiological outcomes

Table 4 summarizes laboratory and cardiometabolic outcomes pre- and post-intervention. LDL showed a main effect of time, decreasing from pre to post (*p* = 0.046). HDL and HbA1c exhibited significant time × group interactions (HDL: *p* < 0.001, q = 0.005; HbA1c: *p* = 0.010, q = 0.026): HDL decreased in CON (*p* = 0.007) but increased in INT (*p* = 0.031), and HbA1c decreased only in INT (*p* = 0.003). TG, CRP, and glucose showed no significant changes. Baseline-adjusted ANCOVA corroborated both effects [HDL: Hedges’ g = 1.73 [95% CI: 0.56 to 2.90]; HbA1c: g = −1.28 [95% CI: −2.41 to −0.16]] (Table 4). CRP could not be statistically modeled due to a floor effect: 38 of 40 available measurements (95%) were at or below the assay’s detection limit of 0.4 mg/dL, with only two values exceeding it. CRP was therefore reported descriptively only, as median [IQR] with the number of values above the detection limit in each cell (Table 4).

**Table 4:** Cardiometabolic and inflammatory blood markers before (pre) and after (post) the intervention in the control (CON) and intervention (INT) groups.

Outcome	CON (*n* = 9)		INT (*n* = 11)		*p*-values from LMM			*p*-values from ANCOVA	Adj. between-group difference in change [95% CI]	Hedges’ g [95% CI]
	Pre	Post	Pre	Post	Time	Group	Time × Group			
Triglycerides [mg/dl]	124 ± 46.5	124 ± 55.4	147 ± 51.8	132 ± 55.4	0.395	0.480	0.486	0.567	−11.5 [−53.5, 30.5]	−0.38 [−1.38, 0.61]
LDL [mg/dl]	115 ± 44.9	110 ± 41.2	123 ± 45.6	110 ± 35.9	**0**.**036**	0.832	0.450	0.338	−6.89 [−21.8, 8.02]	−0.49 [−1.49, 0.51]
HDL [mg/dl]	72.8 ± 19.6	67.3 ± 19.2**	65.8 ± 18.3	70.2 ± 17.7*	0.709	0.811	**0**.**005**	**0**.**004**	9.47 [3.64, 15.3]	1.73 [0.56, 2.90]
CRP [mg/dl]	0.4 [0.4, 0.4] 1 > LOD	0.4 [0.4, 0.4] 0 > LOD	0.4 [0.4, 0.4] 1 > LOD	0.4 [0.4, 0.4] 0 > LOD	-	-	-	-	-	-
Glucose [mg/dl]	123 ± 13.4	124 ± 11.4	117 ± 8.96	118 ± 12.4	0.816	0.217	0.963	0.600	−2.92 [−14.7, 8.83]	−0.02 [−1.03, 0.99]
HbA1c [mmol/mol]	41.2 ± 5.15	41.4 ± 4.27	40.9 ± 6.03	39.6 ± 6.39**	**0**.**030**	0.685	**0**.**026**	**0**.**017**	−1.39 [−2.49, −0.29]	−1.28 [−2.41, −0.16]

Values are presented as mean ± SD. LDL, low-density lipoprotein; HDL, high-density lipoprotein; CRP, c-reactive protein; HbA1c, glycated haemoglobin; ANCOVA, analysis of covariance; CI, confidence interval; LMM, linear mixed model; LOD, assay detection limit (0.4 mg/dL). Significant difference vs. Pre within the group. Time × group interaction *p*-values are adjusted for multiple comparisons within this outcome family using the Benjamini-Hochberg procedure. The adjusted mean between-group difference in change [95% CI] is from a baseline-adjusted ANCOVA, conducted as a sensitivity analysis estimating the same between-group contrast. Hedges’ g [95% CI] is a bias-corrected standardized effect size for the between-group contrast. CRP values are reported as median [Q1, Q3] with the number of values above the detection limit (>LOD) in each cell; CRP could not be modeled due to a floor effect.

* *p* < 0.05

** *p* < 0.01

Bold values indicate a statistically significant within-group Pre-to-Post difference (*p* < 0.05).

Table 5 summarizes cardiologic outcomes pre- and post-intervention. After Benjamini-Hochberg correction for multiple comparisons within the ECG and echocardiography families, none of the observed time × group interactions remained statistically significant. The QT showed a main effect of group (*p* = 0.039), with lower values in INT across time (*p* = 0.048), and no effects of time or interaction. TTE analysis similarly showed nominally significant time  ×  group interactions for LVOT and interventricular septum diameter in diastole (IVSd), but neither remained significant after correction for multiple comparisons (Table 5).

**Table 5:** Cardiologic outcomes before (pre) and after (post) the intervention in the control (CON) and intervention (INT) groups. .

Outcome	CON (*n* = 9)		INT (*n* = 11)		*p*-values from LMM			*p*-values from ANCOVA	Adj. between-group difference in change [95% CI]	Hedges’ g [95% CI]
	Pre	Post	Pre	Post	Time	Group	Time × Group			
HR [bpm]	67.6 ± 9.85	68.4 ± 9.54	71.7 ± 8.55	65.7 ± 6.40	0.106	0.829	0.100	0.087	−5.13 [−11.1, 0.82]	−0.93 [−1.89, 0.02]
RRsys [mmHg]	136 ± 15.4	138 ± 17.6	139 ± 10.6	130 ± 9.11	0.196	0.633	0.142	0.101	−8.80 [−19.5, 1.89]	−0.78 [−1.72, 0.16]
RRdia [mmHg]	83.2 ± 6.78	85.7 ± 14.0	90.3 ± 12.0	82.5 ± 10.6	0.213	0.652	0.100	0.079	−8.63 [−18.4, 1.12]	−1.01 [−1.98, −0.05]
QT [ms]	402 ± 21.1	402 ± 22.5	387 ± 18.9	384 ± 25.2	0.764	**0**.**039**	0.790	0.282	−12.7 [−36.7, 11.4]	−0.12 [−1.02, 0.79]
QRS [ms]	95.4 ± 11.5	95.6 ± 10.9	96.7 ± 13.0	94.6 ± 13.8	0.207	0.973	0.243	0.203	−2.18 [−5.64, 1.29]	−0.59 [−1.52, 0.33]
PQ [ms]	183 ± 37.0	182 ± 32.4	181 ± 20.8	179 ± 17.7	0.576	0.833	0.790	0.722	−1.77 [−12.1, 8.55]	−0.11 [−1.02, 0.80]
LVOT [cm]	19.9 ± 1.45	20.4 ± 1.01	19.9 ± 1.92	19.4 ± 1.43	0.983	0.387	0.116	0.011	−1.09 [−1.90, −0.29]	−0.93 [−1.89, 0.02]
LVIDd [cm/m^2^]	42.2 ± 4.24	42.4 ± 3.85	40.3 ± 4.73	40.8 ± 3.57	0.219	0.327	0.599	0.864	−0.09 [−1.12, 0.95]	0.22 [−0.69, 1.13]
LVM [g]	82.2 ± 19.3	82.3 ± 18.9	69.6 ± 13.8	70.2 ± 12.8	0.273	0.088	0.559	0.821	−0.16 [−1.63, 1.31]	0.24 [−0.66, 1.15]
Aorta [mm]	32.6 ± 2.51	32.9 ± 2.57	31.4 ± 2.50	31.3 ± 2.53	0.289	0.205	0.166	0.104	−0.43 [−0.95, 0.10]	−0.78 [−1.72, 0.16]
IVSd [cm]	9.39 ± 2.47	9.96 ± 2.29	9.42 ± 2.26	9.09 ± 1.92	0.535	0.659	0.116	0.025	−0.89 [−1.65, −0.12]	−0.96 [−1.92, −0.00]
LA-Volume [ml/m^2^]	31.3 ± 6.95	31.3 ± 6.64	29.6 ± 6.83	30.6 ± 6.40	0.293	0.692	0.557	0.514	0.71 [−1.53, 2.95]	0.35 [−0.56, 1.26]
Ejection fraction [%]	56.1 ± 4.70	56.7 ± 6.63	57.5 ± 4.59	62.1 ± 4.28	**0**.**037**	0.076	0.168	0.057	4.67 [−0.15, 9.50]	0.71 [−0.22, 1.65]

Values are presented as mean ± SD. RRsys, systolic blood pressure; RRdia, diastolic blood pressure; LVOT, left ventricular outflow tract diameter; LVIDd, left ventricular internal diameter in diastole; LVM, left ventricular mass; IVSd, interventricular septum diameter in diastole; LA, left atrium; ANCOVA, analysis of covariance; CI, confidence interval; LMM, linear mixed model. Time × group interaction *p*-values are adjusted for multiple comparisons within this outcome family using the Benjamini-Hochberg procedure. The adjusted mean between-group difference in change [95% CI] is from a baseline-adjusted ANCOVA, conducted as a sensitivity analysis estimating the same between-group contrast. Hedges’ g [95% CI] is a bias-corrected standardized effect size for the between-group contrast.

## 4 Discussion

The principal finding of the present study is that a brief, twice-weekly low-load cycling program combined with individualized BFR elicited clinically meaningful improvements in functional mobility in healthy adults over 70 years of age. Over the six-week intervention, the baseline-adjusted between-group difference in DEMMI score was 6.64 points (95% CI: 1.28 to 12.00; *p* = 0.018). Health-related QoL, the second co-primary outcome, improved significantly in INT relative to CON across several SF-36 domains, including vitality [adjusted mean difference = 6.94 points (95% CI: 0.29 to 13.59), *p* = 0.042] and mental health (*p* < 0.001). Among secondary outcomes, INT additionally showed significant gains in lower-limb strength (leg press and leg extension, both *p* < 0.05) and body cell mass (+4.1%), non-significant but moderate-to-large effects on TUG and 6MWT, and favorable secondary exploratory changes in HDL cholesterol and HbA1c (both *p* < 0.05). To the best of the authors`knowledge, this is the first study showing that even a deliberately low-dose exercise intervention can induce functionally meaningful adaptations when combined with individualized BFR.

To better characterize the study population, participants were compared with established normative reference values. At baseline, grip strength (48) and leg press 1RM values (49) were generally below age- and sex-specific reference norms, indicating reduced physical performance despite independent community living. Nevertheless, according to the European Working Group on Sarcopenia in Older People (EWGSOP2), none of the participants fulfilled the diagnostic criteria for sarcopenia, as all exceeded the recommended cut-off values for grip strength, TUG performance, and gait speed (50).

These findings suggest that the cohort represents older adults with early age-related functional decline rather than established sarcopenia, a population in whom preventive exercise interventions are particularly relevant. Consistent with this interpretation, four participants (two in each group) were unable to complete 400 m during the 6MWT at baseline, whereas after the intervention only one participant in the BFR group remained below this threshold. Therefore, the present cohort reflects a clinically relevant population of community-dwelling older adults with reduced physical performance but preserved functional independence, emphasizing the potential value of time-efficient interventions aimed at maintaining mobility before overt disability develops. As the cohort was predominantly female (14 of 20 participants), community-dwelling, and free of sarcopenia by EWGSOP2 criteria, the present findings apply to relatively healthy older adults with early functional decline and should not be extrapolated to sarcopenic, frail, or institutionalized populations without further study.

### 4.1 Effects on functionality, balance & quality of life

The principal finding of the present study is that a brief, twice-weekly low-load cycling program combined with individualized BFR elicited clinically meaningful improvements in functional mobility in healthy adults over 70 years of age. Despite a total exercise exposure of only twelve supervised 15-minute sessions, DEMMI scores increased by 8.3 points in INT (Pre: 85.2 ± 13.4; Post: 93.5 ± 9.45), compared with a smaller, non-significant increase in CON (Pre: 86.2 ± 12.8; Post: 87.6 ± 10.4). The baseline-adjusted between-group difference was 6.64 points (95% CI: 1.28 to 12.00), closely approaching the minimal clinically important difference (MCID) of 7 points reported in healthy, community-dwelling older adults (*n* = 61, mean age 76.5 ± 5.5 years) using a distribution-based method (51). These findings suggest that even a deliberately low-dose exercise intervention can induce functionally meaningful adaptations when combined with individualized BFR.

To the best of our knowledge, this is the first RCT to evaluate functional mobility following endurance-based BFR training using the DEMMI, a validated instrument for assessing mobility and predicting functional status in older adults (18). Previous literature has not consistently shown statistically significant advantages of BFR training on QoL or physical performance outcomes in older adults compared with other exercise interventions (17). Notably, only a single END + BFR study contributed to these earlier analyses, illustrating the underrepresentation of endurance exercise trials in older adults.

Additionally, both groups improved on the 6MWT (INT: +9%; CON: +1.9%) and TUG performance (INT: −10.3%; CON: −5.6%), reflecting significant main effects of time without a significant time  ×  group interaction. Nevertheless, the estimated between-group effect sizes for both outcomes were of moderate-to-large magnitude [6MWT: Hedges’ g = 0.79 [95% CI: −0.15 to 1.73]; TUG: g = −0.68 [95% CI: −1.61 to 0.25]], with the wide confidence intervals reflecting limited statistical power rather than an absence of a treatment effect. The magnitude of change observed in INT falls within the range reported for walking-based BFR interventions. Meta-analytic evidence across 8 RCTs confirms this direction of effect for TUG, with BFR training associated with a significant improvement in TUG time compared to non-BFR conditions (mean difference = −0.71 s, 95% CI: −1.05 to −0.37, *p* < 0.001) (52). Similar reductions in TUG time have been reported individually by Ozaki et al. (−10.7%) (12), while increases in 6MWT performance have been described by Clarkson et al. (+9%) (11). Remarkably, these functional improvements were achieved despite a substantially lower training frequency (two sessions per week) than previous walking-based BFR interventions, highlighting the potential of individualized BFR to maximize training efficiency in older adults.

The functional improvements were paralleled by significant increases in several SF-36 domains, including physical functioning, vitality, mental health, social functioning, and general health perception (Table 3). These findings suggest that the observed gains in mobility translated into meaningful improvements in participants’ perception of their physical and mental well-being. These improvements in perceived physical fitness and vitality are consistent with findings by Zhang and colleagues in sarcopenic patients following a 12-week resistance training program (53). However, while the authors did not observe any significant differences between LL-BFR-RT and conventional HL-RT under free-flow conditions, the present results suggest that, despite no significant between-group difference in mean external training power (INT: 35.1 ± 12.4 W vs. CON: 40.8 ± 13.3 W; *p* = 0.333), participants in INT reported significantly higher perceived exertion than CON (*p* = 0.020). This finding is consistent with the known effect of BFR in increasing perceived effort even without a statistically significant difference in external workload. However, given the small sample size and the absence of a formal equivalence test for training workload, this should be regarded as a hypothesis-generating observation for future, adequately powered trials, rather than confirmed evidence of a BFR-specific physiological stimulus underlying the functional and quality-of-life adaptations observed in INT. The vitality domain showed a marked baseline imbalance, with INT scoring significantly lower than CON before the intervention (*p* = 0.012). Regression to the mean is therefore a plausible competing explanation for part of the apparent improvement in INT: groups that score unusually low at baseline tend to move toward the population average on remeasurement regardless of any true intervention effect. Baseline-adjusted ANCOVA, which is more robust to this bias than an unadjusted change-score comparison, still showed a statistically significant between-group difference [adjusted mean difference = 6.94 points (95% CI: 0.29 to 13.59), *p* = 0.042]. However, ANCOVA does not fully eliminate regression-to-the-mean bias, and given the baseline imbalance and modest sample size, this finding should be interpreted with caution. Using the benchmark of approximately 0.5 SD as a minimally important difference for health-related quality of life measures (54), the observed effect sizes for vitality (g = 1.56) and mental health (g = 1.61) clearly exceed this threshold, indicating a magnitude of change likely to be clinically meaningful to patients, beyond mere statistical significance.

The ability to achieve clinically meaningful functional improvements after only twelve supervised exercise sessions may be explained by the enhanced physiological stimulus induced by BFR. Compared with identical low-load exercise performed under free-flow conditions, BFR increases metabolic stress, muscle fiber recruitment, and perceived exertion despite identical external workloads (11) (55),. Understanding the accompanying neuromuscular adaptations is therefore essential to explain how such a deliberately low exercise dose translated into clinically meaningful improvements in mobility.

### 4.2 Effects on skeletal muscle morphology and function

The functional improvements observed in the present study were accompanied by marked increases in lower-limb muscle strength and mass, suggesting that individualized BFR amplified the hypertrophy stimulus despite the low external training load. Previous investigations in young, healthy adults have demonstrated that short LL-END combined with BFR can induce substantial improvements in muscular and aerobic outcomes. Specifically, Abe et al. showed that 15 min of low-intensity cycling with BFR (40% VO₂max, fixed cuff pressure progressively increased from 160 to 210 mmHg) elicited superior gains in VO₂max, time to exhaustion, muscle hypertrophy, and maximal strength compared with 45 min of conventional endurance exercise at matched intensities (15). In comparison to high-load resistance training (HL-RT) (4 × 10 repetitions, 70% 1RM), Conceição et al. (56) reported that a 15-minute END + BFR exercise induced inferior, however meaningful enhancements (significantly higher than LL-END alone) in muscle strength (END + BFR ∼10%; HL-RT ∼35% in RM) and thigh muscle cross-sectional area (END + BFR ∼10%; HL-RT ∼12% in RM) following an eight-week intervention. However, these studies were conducted in young populations with higher training frequency and longer intervention duration, limiting their transferability to older adult cohorts.

In contrast, the present study demonstrates that a comparatively brief, outpatient BFR-cycling protocol performed twice weekly over six weeks is sufficient to induce significant strength gains in adults older than 70 years. The addition of BFR to the low-intensity cycling exercise obtained improvements of around 23% in both leg press and leg extension strength, whereas the control condition showed minimal or even negative changes (+6%; −4%). These improvements are superior to findings in the literature, applying low-load BFR resistance training (LL-BFR-RT) exercises (17% increase in leg press 1RM) (57) or after BFR-walking exercise (5.9% increase in maximal isometric strength) (12), despite differences in intervention frequency and duration.

Notably, handgrip strength declined significantly in CON while remaining stable in INT. Individual trajectories indicate that this decline was driven predominantly by two of nine CON participants, with comparatively minor changes in the remainder. Given that the minimum detectable change for handgrip dynamometry in older adults has been reported to range widely (14.5–98.5% of baseline values) depending on protocol and population (58), and no study-specific reliability estimate was obtained for our testing protocol, this decline cannot be reliably distinguished from measurement variability in a small sample. We therefore do not interpret this finding as evidence of a protective effect of lower-limb BFR training on non-trained muscle groups. Additionally, participants of the intervention group demonstrated a significant increase in total exercise time during the incremental cycling test in comparison to the control group (Figure 3), whereas no statistical difference in workload at a blood lactate concentration of 2 mmol·L⁻¹ (W/2 mmol) was observed. These findings suggest that END + BFR primarily enhanced exercise tolerance rather than inducing measurable improvements in submaximal aerobic performance within the relatively short intervention period. However, beyond strength and endurance capacity, previous BFR walking studies in older populations demonstrated supportive enhancements in thigh muscle cross-sectional area ranging from 3%–6% (12, 13). Consistent with these reports, the present study observed a significant increase in body cell mass (+4.1%) exclusively in the BFR group, although the magnitude was smaller than in younger cohorts, likely reflecting age-related anabolic resistance and differences in assessment methodology. Of note, total body water increased to a similar extent in both groups, with no significant between-group difference, which argues against differential hydration status as an explanation for the group-specific increase in BCM observed in INT.

Notably, cycling-based BFR interventions remain scarcely investigated in older adults, and the few available cycle ergometry studies applying supramaximal cuff pressures cannot be considered representative of classical BFR training paradigms (59). In this context, our results extend existing evidence by demonstrating that clinically feasible, moderate-pressure BFR cycling not only improves endurance capacity but also induces robust strength and muscle mass adaptations in an older adult cohort, while also highlighting the persistent underrepresentation of endurance-based BFR protocols in the literature.

### 4.3 Safety of blood flow restriction training in older adults

Unlike previous BFR studies in older adults, the present trial combined functional efficacy outcomes with comprehensive cardiovascular, metabolic, and laboratory-based safety assessments. Over the six-week intervention period, no dropouts, adverse events, or clinically relevant side effects were observed, while participant compliance with the BFR protocol was high, underscoring the feasibility of individualized ambulatory END + BFR training in healthy older adults (60, 61).

Cardiovascular safety was rigorously evaluated using ECG, TTE, and blood pressure measurements, at rest before and after the intervention period. No adverse effects on cardiac rhythm or ECG parameters were detected. Transthoracic echocardiographic analyses showed time  ×  group interactions for IVSd and LVOT that did not reach statistical significance in *post-hoc* contrasts (*p* ≥ 0.068), alongside a non-significant reduction in resting blood pressure. Ejection fraction (EF) showed a significant main effect of time, with higher values after the intervention in both groups (Table 5), indicating a time-related rather than BFR-specific effect. These findings should be interpreted as showing no adverse effects on cardiovascular function rather than as statistic evidence of a BFR-induced structural cardiac benefit.

For context, some prior work in other populations has reported favorable cardiac adaptations following BFR training: Kim et al. reported improved cardiac structure and diastolic function in runners with exercise-induced hypertension (62), and Wu and Liu reported improvements in cardiac function parameters, exercise capacity, and QoL in patients with heart failure with reduced ejection fraction following combined BFR and isometric exercise training (63). Reverse myocardial remodeling therefore remains a plausible but unconfirmed mechanistic hypothesis for BFR-related cardiac adaptations in general, which the present non-significant echocardiographic findings can neither confirm nor rule out. A systematic review and meta-analysis in older adults similarly reported favorable effects of low-load BFR training on hemodynamic responses and vascular function (64).

Several outcomes showed no significant time  ×  group interaction and merit brief discussion rather than silence. TUG [g = −0.68 (95% CI: −1.61 to 0.25)] and 6MWT [g = 0.79 (95% CI: −0.15 to 1.73)] showed moderate-to-large point estimates favoring INT despite wide, null-spanning confidence intervals, consistent with insufficient power rather than a true absence of effect. Submaximal aerobic performance (W/2 mmol) likewise showed no significant between-group difference [adjusted mean difference = −8.25 W [95% CI: −31.7 to 15.2]; g = −0.59 [95% CI: −1.76 to 0.57]]. Body water (*p* = 0.615), body fat [*p* = 0.139, g = −0.70 (95% CI: −1.64 to 0.23)], and extracellular mass [*p* = 0.296, g = 0.48 (95% CI: −0.44 to 1.39)] also showed no significant between-group differences, suggesting the intervention selectively affected body cell mass without broader compositional change. Echocardiographic parameters (EF, IVSd, LVOT) similarly showed no significant interactions after correction for multiple comparisons (*p* ≥ 0.068). Collectively, these findings suggest adaptations relatively selective to functional mobility, QoL, strength, and body cell mass, without detectable broader change in aerobic capacity, body composition, or cardiac structure within this timeframe.

From a metabolic perspective, BFR training elicited significant improvements in both glucose and lipid metabolism, as reflected by reduced HbA1c levels and increased HDL cholesterol concentrations (Table 4). To contextualize the magnitude of these changes: the observed increase in HDL cholesterol [adjusted between-group difference = 9.47 mg/dL (95% CI: 3.64 to 15.3)] exceeds, several-fold, the increment associated with meaningful cardiovascular risk reduction in prospective epidemiological cohorts (approximately 2%–3% relative risk reduction per 1 mg/dL increase) (65). However, this extrapolation from population-level associations does not establish that the present exercise-induced increase confers an equivalent clinical benefit. In contrast, the observed reduction in HbA1c [adjusted between-group difference = −1.39 mmol/mol (95% CI: −2.49 to −0.29)] falls below the 3 mmol/mol (0.3 percentage point) threshold generally considered clinically meaningful for reducing long-term diabetic complications in populations with prediabetes (66). While statistically significant, this change is therefore best interpreted as a favorable metabolic trend rather than an established clinical benefit, particularly in this non-diabetic population. These findings are consistent with previous reports in older adults with type 2 diabetes and sarcopenia, as well as in younger obese populations (53, 67, 68). A comparable direction of lipid improvement, including increased HDL cholesterol, has recently been reported following a 10-week walking-based training program without BFR in obese postmenopausal women (69), suggesting that at least part of the metabolic benefit observed here may be attributable to the aerobic training stimulus itself rather than to BFR specifically. While mechanisms were not directly measured in the present study, BFR-induced improvements in glucose metabolism have been attributed to enhanced skeletal muscle glucose uptake via increased GLUT4 expression and translocation, potentially mediated by metabolic stress–induced activation of AMP-activated protein kinase (AMPK) (70, 71). Improvements in lipid metabolism are likely driven by indirect training effects such as increased muscle mass, enhanced insulin sensitivity, and improved metabolic flexibility, with BFR allowing these adaptations to occur at substantially lower mechanical loads (72).

Taken together, the present results indicate that, in this small, healthy, and closely supervised sample, individualized moderate-pressure BFR cycling using a validated pressure-measurement device was well tolerated at rest over six weeks, without clinically relevant adverse cardiovascular or metabolic findings; this should not be read as an established safety profile of BFR training for older adults in general, which would require larger, prospectively registered trials with monitoring during exercise itself. The combination of favorable metabolic adaptations and the absence of adverse resting cardiovascular findings supports further investigation of BFR training into preventive and rehabilitative exercise programs for older adults, particularly in populations for whom high mechanical loading is not suitable or even contraindicated.

## 5 Conclusion

The present study demonstrates that a brief, twice-weekly 15-minute low-load cycling program combined with individualized BFR is sufficient to induce significant rises in muscle strength, with an accompanying between-group improvement in functional mobility (DEMMI) that closely approached the established minimal clinically important difference, indicating that the intervention plausibly translated into functionally relevant benefits despite the intentionally minimal exercise dose. No dropouts, adverse events, or clinically relevant adverse findings on resting cardiovascular and metabolic monitoring were observed during the six-week training period in this small, supervised sample, supporting short-term feasibility. However, broader conclusions about cardiovascular safety during BFR exercise itself, which was not systematically monitored here, require confirmation in larger trials. Two practical implications follow. First, for older adults unable or unwilling to perform conventional high-load resistance training, such a program, delivered by a physiotherapist or sports therapist trained in BFR application, using individualized cuff pressure set at 60% of measured LOP and an individually prescribed relative training intensity, twice weekly for a minimum of six weeks, may represent a time-efficient option within preventive or rehabilitative exercise settings. Second, given that the observed between-group effect and the established minimal clinically important difference for the DEMMI were of comparable magnitude, we suggest DEMMI reassessment at the end of a six-week block as a practical, evidence-based indicator of whether the intervention is producing a clinically meaningful functional response in an individual patient.

### 5.1 Limitations

Several limitations of the present study should be acknowledged when interpreting these findings:

1. The present study included a relatively small sample size (*N* = 20). As no pilot data were available to power the co-primary outcomes (DEMMI and health-related quality of life) at the time of protocol design, the sample size calculation was instead based on published effect sizes for the secondary muscular strength outcomes. While this provides adequate power to detect large effects on muscle strength, it affords only limited precision for the primary DEMMI and quality-of-life findings, and the remaining secondary and exploratory outcomes (TUG, 6MWT, body composition, endurance, and metabolic and cardiological parameters) should be interpreted with corresponding caution. As no formal adjustment for multiplicity was made between the two co-primary outcomes, the DEMMI and quality-of-life findings should likewise be interpreted jointly rather than as independent confirmatory results.
2. Despite randomization, groups showed a numerically large, though not statistically significant, baseline difference in handgrip strength. A baseline imbalance of this magnitude in a small sample can inflate a time  ×  group interaction even without reaching formal significance. We therefore also report the baseline-adjusted ANCOVA effect for handgrip strength, which confirms the effect after accounting for this baseline difference.
3. The trial was not prospectively registered in a public registry, which limits transparency regarding pre-specified outcomes and analyses.
4. While the outcome assessor was blinded to group allocation, participant blinding could not be fully guaranteed given the perceptible difference in cuff pressure between INT (121 ± 14 mmHg) and the CON sham condition (20 mmHg). As a single rater performed all DEMMI assessments, inter-rater reliability could not be evaluated, intra-rater reliability was not formally assessed and in this study. Additionally, no formal post-intervention blinding check was performed, so the extent to which incomplete participant blinding may have influenced self-reported outcomes, particularly health-related quality of life, cannot be quantified. No study-specific reliability estimate was obtained for the handgrip dynamometry protocol used in this study, and reported minimum detectable change values for handgrip strength in older adults vary widely across the literature (58). This limits confidence in interpreting individual-level changes in this outcome.
5. The large number of outcome families tested (functional, quality-of-life, body-composition, metabolic, ECG, and echocardiographic) increases the risk of Type I error; Bonferroni correction was applied only within individual *post-hoc* comparisons and not across the broader set of outcome families, so secondary and exploratory findings should be interpreted with appropriate caution.
6. Body composition was assessed using bioelectrical impedance analysis rather than direct imaging techniques.
7. Exercise intensity was individualized using an estimated maximal heart rate (220−age), a formula with considerable error in older adults. Heart-rate-limiting medication, including beta-blockers and other antihypertensive drugs, was not systematically documented and could therefore not be accounted for in the intensity prescription. Furthermore, echocardiographic parameters (ejection fraction, interventricular septum diameter in diastole, and left ventricular outflow tract diameter) were analyzed by a single blinded investigator, and intra- or inter-observer reliability was not formally assessed; the non-significant time  ×  group interactions reported for these parameters should therefore be interpreted with this measurement uncertainty in mind.
8. Cardiovascular assessments were performed at rest before and after the intervention. The specific stopping criterion (volitional exhaustion, heart rate threshold, or loss of cadence) that terminated the incremental cycling test was not systematically recorded for each participant and timepoint. Furthermore, acute cardiovascular responses during the BFR-cycling sessions themselves were not systematically monitored, so the present findings support short-term feasibility and the absence of adverse resting cardiovascular changes rather than establishing comprehensive cardiovascular safety.
9. Outcomes were assessed only immediately after the six-week intervention, and no follow-up assessment was performed to evaluate the durability of the observed adaptations.
10. The heterogeneity of BFR methodologies and the lack of protocol standardization across studies limit direct comparability with the existing literature.

Consequently, future research should prioritize prospectively registered, adequately powered trials using well-established devices and standardized BFR protocols, report effect sizes with confidence intervals, and include longer-term follow-up. In addition, future studies should clearly specify the methodology and apparatus used to enhance transparency and reproducibility.

## 6 Transparency declaration

The authors affirm that this manuscript is an honest, accurate, and transparent account of the study being reported; that no important aspects of the study have been omitted; and that any discrepancies from the study as planned have been explained.

## Data Availability

The original contributions presented in the study are included in the article/Supplementary Material, further inquiries can be directed to the corresponding author/s.

## Supplementary material

Supplementary Figure S1 Power output (A) and Rating of perceived exertion (RPE) (B) measured across the training session. Thin lines represent individual participants; thick lines represent the group mean (CON = control group, INT = intervention group). Group values are mean ± SD (A) or median ± IQR (B); between-group comparisons use independent-samples t-tests (A) or the Wilcoxon rank-sum test (B, given the ordinal nature of RPE).
