# Supplementary material for: Effects of individualized blood flow restriction during brief twice-weekly low-load cycling on mobility and muscle function in adults older than 70 years
Source: Front Sports Act Living. 2026 Oct 2;8:1933001. doi: 10.3389/fspor.2026.1933001 (PMC13672636; doi:10.3389/fspor.2026.1933001)
Supplement: Supplementary file 1 [file Table1.docx]

**Supplementary Table. Overview of outcome measures, instruments, original developer references, units, and reliability evidence.**

| **Variable** | **Instrument / Device** | **Original developer reference** | **Unit** | **Reliability evidence** |
| --- | --- | --- | --- | --- |
| Functional mobility (primary) | De Morton Mobility Index (DEMMI), German version | de Morton NA, Davidson M, Keating JL. Health Qual Life Outcomes (2008) 6:63. | points (0–100, Rasch-interval) | German version: inter-rater ICC = 0.94 (Braun et al., 2015, BMC Geriatr). Not reassessed in this study; a single blinded rater performed all DEMMI assessments, so inter-/intra-rater reliability could not be evaluated here. |
| Health-related quality of life (primary) | SF-36 Health Survey, German version | Ware JE, Sherbourne CD. Med Care (1992) 30:473–483. | points (0–100 per domain) | German translation/validation: Bullinger, 1995, Soc Sci Med. Not reassessed in this study. |
| Dynamic balance / functional mobility (secondary) | Timed Up and Go (TUG) test | Podsiadlo D, Richardson S. J Am Geriatr Soc (1991) 39:142–148. | seconds | Not separately assessed in this study; test–retest reliability established in the general literature (see ref. cited in Methods). |
| Walking capacity (secondary) | Six-Minute Walk Test (6MWT), 20-m corridor | Holland AE et al. (ATS/ERS Technical Standard) Eur Respir J (2014) 44:1428–1446. | meters | Familiarization trial performed at baseline (T0) only; single trial at each assessment thereafter. No reliability formally computed in this study. |
| Lower-limb strength (secondary) | One-repetition maximum (1RM), leg press & leg extension (proxomed functional stem / leg extensor) | Barbalho M et al. Sports (Basel) (2018) 6:171. | kg | Reproducibility and validity of the 1RM protocol reported in Barbalho et al., 2018. |
| Handgrip strength (secondary) | Baseline Hydraulic Hand Dynamometer (Fabrication Enterprises, Inc., White Plains, NY, USA) | Mathiowetz V et al. (standardized position) J Hand Surg Am (1984) 9:222–226. | kg | Mean of 3 trials (~1–2 min rest between trials) used for analysis. No formal reliability assessed in this study. |
| Body composition (BCM, body water, fat mass) | seca mBCA 515, 8-point BIA (seca GmbH & Co. KG, Hamburg, Germany) | Bosy-Westphal A et al. Eur J Clin Nutr (2013) 67(Suppl 1):S14–S21. | kg / % | Device validated against 4-compartment model (device-level validation). Not reassessed in this study. |
| Endurance capacity (PWC130, total time; W/2mmol) | Incremental cycling ergometer test (Thera-trainer tigo, medica Medizintechnik GmbH, Hochdorf, Germany); capillary lactate: BIOSEN S-line (EKF-diagnostic GmbH, Barleben, Germany) | Institution-specific protocol; no external developer reference. | minutes / Watts | Stopping criterion (exhaustion, HR>130, cadence loss) not recorded per participant (see Limitations); no formal reliability assessed. |
| Cardiac structure & function | Transthoracic echocardiography (TTE), Philips EPIQ (Philips, Amsterdam, The Netherlands) | — | EF: %; IVSd, LVOT: mm | Single blinded reader; intra-/inter-observer reliability not formally assessed (see Limitations). |
| Laboratory parameters (HbA1c, HDL, LDL, TG, CRP, glucose) | Clinic central laboratory (Zentrallabor) | — | mg/dL, mmol/mol, mg/L | Standard clinical laboratory quality control; not study-specific reliability data. |
