# Supplementary material for: Effects of individualized blood flow restriction during brief twice-weekly low-load cycling on mobility and muscle function in adults older than 70 years
Source: Front Sports Act Living. 2026 Oct 2;8:1933001. doi: 10.3389/fspor.2026.1933001 (PMC13672636; doi:10.3389/fspor.2026.1933001)
Supplement: Supplementary file 2 [file Table2.docx]

**Supplement Table 2.** Full linear mixed model output for all outcomes: fixed-effect estimates, standard errors, Satterthwaite-approximated degrees of freedom, t-values, and p-values for each model term (Intercept, Time, Group, Time × Group).

| **Category** | **Variable** | **Term** | **Estimate** | **SE** | **df** | **t** | **p** |
| --- | --- | --- | --- | --- | --- | --- | --- |
| Functional and balance outcomes | 6MWT [m] | Intercept | 502.56 | 27.54 | 23.6 | 18.25 | <0.001 |
|  | 6MWT [m] | Time (Post vs Pre) | 3.22 | 15.78 | 20.0 | 0.20 | 0.840 |
|  | 6MWT [m] | Group (INT vs CON) | -3.92 | 37.13 | 23.6 | -0.11 | 0.917 |
|  | 6MWT [m] | Time x Group | 41.10 | 21.28 | 20.0 | 1.93 | 0.068 |
|  | 6MWT [km/h] | Intercept | 5.03 | 0.27 | 24.5 | 18.39 | <0.001 |
|  | 6MWT [km/h] | Time (Post vs Pre) | 0.03 | 0.17 | 20.0 | 0.18 | 0.855 |
|  | 6MWT [km/h] | Group (INT vs CON) | 0.02 | 0.37 | 24.5 | 0.04 | 0.967 |
|  | 6MWT [km/h] | Time x Group | 0.23 | 0.24 | 20.0 | 0.98 | 0.337 |
|  | TUG [s] | Intercept | 7.06 | 0.43 | 21.9 | 16.26 | <0.001 |
|  | TUG [s] | Time (Post vs Pre) | -0.39 | 0.19 | 20.0 | -2.10 | 0.049 |
|  | TUG [s] | Group (INT vs CON) | 0.27 | 0.59 | 21.9 | 0.47 | 0.644 |
|  | TUG [s] | Time x Group | -0.42 | 0.25 | 20.0 | -1.66 | 0.113 |
|  | DEMMI [points] | Intercept | 86.22 | 3.67 | 24.4 | 23.49 | <0.001 |
|  | DEMMI [points] | Time (Post vs Pre) | 1.33 | 2.33 | 20.0 | 0.57 | 0.574 |
|  | DEMMI [points] | Group (INT vs CON) | -1.04 | 4.95 | 24.4 | -0.21 | 0.835 |
|  | DEMMI [points] | Time x Group | 7.03 | 3.14 | 20.0 | 2.24 | 0.037 |
| Quality of life (SF-36) | Physical Functioning | Intercept | 85.56 | 4.24 | 26.1 | 20.15 | <0.001 |
|  | Physical Functioning | Time (Post vs Pre) | 6.67 | 3.43 | 19.0 | 1.94 | 0.067 |
|  | Physical Functioning | Group (INT vs CON) | -6.06 | 5.85 | 26.1 | -1.03 | 0.310 |
|  | Physical Functioning | Time x Group | 6.33 | 4.73 | 19.0 | 1.34 | 0.196 |
|  | Role Physical | Intercept | 100.00 | 3.81 | 38.0 | 26.23 | <0.001 |
|  | Role Physical | Time (Post vs Pre) | -5.56 | 5.39 | 38.0 | -1.03 | 0.309 |
|  | Role Physical | Group (INT vs CON) | -10.00 | 5.26 | 38.0 | -1.90 | 0.065 |
|  | Role Physical | Time x Group | 15.56 | 7.43 | 38.0 | 2.09 | 0.043 |
|  | Bodily Pain | Intercept | 90.67 | 3.71 | 35.7 | 24.43 | <0.001 |
|  | Bodily Pain | Time (Post vs Pre) | 5.78 | 4.53 | 19.0 | 1.27 | 0.218 |
|  | Bodily Pain | Group (INT vs CON) | -6.07 | 5.12 | 35.7 | -1.19 | 0.243 |
|  | Bodily Pain | Time x Group | 9.62 | 6.25 | 19.0 | 1.54 | 0.140 |
|  | General Health | Intercept | 73.11 | 2.48 | 26.9 | 29.51 | <0.001 |
|  | General Health | Time (Post vs Pre) | 0.56 | 2.10 | 19.0 | 0.26 | 0.794 |
|  | General Health | Group (INT vs CON) | -7.11 | 3.42 | 26.9 | -2.08 | 0.047 |
|  | General Health | Time x Group | 7.34 | 2.90 | 19.0 | 2.54 | 0.020 |
|  | Vitality | Intercept | 69.44 | 3.20 | 22.4 | 21.69 | <0.001 |
|  | Vitality | Time (Post vs Pre) | 5.00 | 1.83 | 19.0 | 2.73 | 0.013 |
|  | Vitality | Group (INT vs CON) | -13.44 | 4.41 | 22.4 | -3.05 | 0.006 |
|  | Vitality | Time x Group | 9.50 | 2.52 | 19.0 | 3.77 | 0.001 |
|  | Social Functioning | Intercept | 90.28 | 5.50 | 22.6 | 16.43 | <0.001 |
|  | Social Functioning | Time (Post vs Pre) | 0.00 | 3.26 | 19.0 | 0.00 | 1.000 |
|  | Social Functioning | Group (INT vs CON) | -11.53 | 7.58 | 22.6 | -1.52 | 0.142 |
|  | Social Functioning | Time x Group | 10.00 | 4.49 | 19.0 | 2.23 | 0.038 |
|  | Role Emotional | Intercept | 100.00 | 6.89 | 36.5 | 14.52 | <0.001 |
|  | Role Emotional | Time (Post vs Pre) | 0.00 | 8.68 | 19.0 | 0.00 | 1.000 |
|  | Role Emotional | Group (INT vs CON) | -10.00 | 9.49 | 36.5 | -1.05 | 0.299 |
|  | Role Emotional | Time x Group | -6.67 | 11.97 | 19.0 | -0.56 | 0.584 |
|  | Mental Health | Intercept | 66.67 | 3.46 | 20.8 | 19.26 | <0.001 |
|  | Mental Health | Time (Post vs Pre) | 4.44 | 1.49 | 19.0 | 2.98 | 0.008 |
|  | Mental Health | Group (INT vs CON) | -0.27 | 4.77 | 20.8 | -0.06 | 0.956 |
|  | Mental Health | Time x Group | 7.96 | 2.06 | 19.0 | 3.87 | 0.001 |
|  | PCS | Intercept | 54.31 | 0.93 | 27.5 | 58.38 | <0.001 |
|  | PCS | Time (Post vs Pre) | 1.03 | 0.81 | 19.0 | 1.27 | 0.220 |
|  | PCS | Group (INT vs CON) | -3.39 | 1.28 | 27.5 | -2.64 | 0.013 |
|  | PCS | Time x Group | 4.51 | 1.12 | 19.0 | 4.02 | <0.001 |
|  | MCS | Intercept | 50.58 | 1.88 | 21.4 | 26.90 | <0.001 |
|  | MCS | Time (Post vs Pre) | 1.05 | 0.93 | 19.0 | 1.14 | 0.270 |
|  | MCS | Group (INT vs CON) | -3.01 | 2.59 | 21.4 | -1.16 | 0.259 |
|  | MCS | Time x Group | 1.88 | 1.28 | 19.0 | 1.48 | 0.156 |
|  | Health Transition | Intercept | 2.22 | 0.26 | 32.7 | 8.61 | <0.001 |
|  | Health Transition | Time (Post vs Pre) | 0.33 | 0.28 | 19.0 | 1.18 | 0.252 |
|  | Health Transition | Group (INT vs CON) | 0.48 | 0.36 | 32.7 | 1.34 | 0.188 |
|  | Health Transition | Time x Group | -1.13 | 0.39 | 19.0 | -2.92 | 0.009 |
| Cardiometabolic and inflammatory markers | Triglycerides [mg/dl] | Intercept | 123.56 | 16.38 | 22.2 | 7.54 | <0.001 |
|  | Triglycerides [mg/dl] | Time (Post vs Pre) | 0.11 | 12.10 | 17.0 | 0.01 | 0.993 |
|  | Triglycerides [mg/dl] | Group (INT vs CON) | 23.82 | 23.87 | 22.2 | 1.00 | 0.329 |
|  | Triglycerides [mg/dl] | Time x Group | -15.61 | 17.64 | 17.0 | -0.89 | 0.388 |
|  | LDL [mg/dl] | Intercept | 114.56 | 13.19 | 18.3 | 8.68 | <0.001 |
|  | LDL [mg/dl] | Time (Post vs Pre) | -4.22 | 5.13 | 17.0 | -0.82 | 0.422 |
|  | LDL [mg/dl] | Group (INT vs CON) | 8.32 | 19.23 | 18.3 | 0.43 | 0.670 |
|  | LDL [mg/dl] | Time x Group | -8.53 | 7.48 | 17.0 | -1.14 | 0.270 |
|  | HDL [mg/dl] | Intercept | 72.78 | 5.88 | 17.7 | 12.38 | <0.001 |
|  | HDL [mg/dl] | Time (Post vs Pre) | -5.44 | 1.71 | 17.0 | -3.19 | 0.005 |
|  | HDL [mg/dl] | Group (INT vs CON) | -7.03 | 8.57 | 17.7 | -0.82 | 0.423 |
|  | HDL [mg/dl] | Time x Group | 9.94 | 2.49 | 17.0 | 3.99 | <0.001 |
|  | Glucose [mg/dl] | Intercept | 123.25 | 3.85 | 25.0 | 32.00 | <0.001 |
|  | Glucose [mg/dl] | Time (Post vs Pre) | 0.75 | 3.73 | 16.0 | 0.20 | 0.843 |
|  | Glucose [mg/dl] | Group (INT vs CON) | -6.00 | 5.45 | 25.0 | -1.10 | 0.281 |
|  | Glucose [mg/dl] | Time x Group | -0.25 | 5.28 | 16.0 | -0.05 | 0.963 |
|  | HbA1c [mmol/mol] | Intercept | 41.25 | 1.83 | 16.3 | 22.58 | <0.001 |
|  | HbA1c [mmol/mol] | Time (Post vs Pre) | 0.12 | 0.34 | 16.0 | 0.37 | 0.714 |
|  | HbA1c [mmol/mol] | Group (INT vs CON) | -0.38 | 2.58 | 16.3 | -0.15 | 0.886 |
|  | HbA1c [mmol/mol] | Time x Group | -1.37 | 0.47 | 16.0 | -2.90 | 0.010 |
| Cardiologic outcomes | HR [bpm] | Intercept | 67.56 | 2.71 | 27.9 | 24.92 | <0.001 |
|  | HR [bpm] | Time (Post vs Pre) | 0.89 | 2.24 | 20.0 | 0.40 | 0.695 |
|  | HR [bpm] | Group (INT vs CON) | 4.17 | 3.65 | 27.9 | 1.14 | 0.263 |
|  | HR [bpm] | Time x Group | -6.89 | 3.02 | 20.0 | -2.28 | 0.033 |
|  | RRsys [mmHg] | Intercept | 136.22 | 4.19 | 29.6 | 32.54 | <0.001 |
|  | RRsys [mmHg] | Time (Post vs Pre) | 1.44 | 3.78 | 20.0 | 0.38 | 0.707 |
|  | RRsys [mmHg] | Group (INT vs CON) | 2.41 | 5.65 | 29.6 | 0.43 | 0.672 |
|  | RRsys [mmHg] | Time x Group | -9.72 | 5.10 | 20.0 | -1.91 | 0.071 |
|  | RRdia [mmHg] | Intercept | 83.22 | 3.53 | 28.7 | 23.60 | <0.001 |
|  | RRdia [mmHg] | Time (Post vs Pre) | 2.44 | 3.04 | 20.0 | 0.80 | 0.431 |
|  | RRdia [mmHg] | Group (INT vs CON) | 7.05 | 4.75 | 28.7 | 1.48 | 0.149 |
|  | RRdia [mmHg] | Time x Group | -10.17 | 4.11 | 20.0 | -2.48 | 0.022 |
|  | QT [ms] | Intercept | 402.00 | 6.98 | 36.6 | 57.56 | <0.001 |
|  | QT [ms] | Time (Post vs Pre) | -0.11 | 8.24 | 20.0 | -0.01 | 0.989 |
|  | QT [ms] | Group (INT vs CON) | -15.18 | 9.42 | 36.6 | -1.61 | 0.116 |
|  | QT [ms] | Time x Group | -3.16 | 11.11 | 20.0 | -0.28 | 0.779 |
|  | QRS [ms] | Intercept | 95.44 | 3.95 | 20.8 | 24.16 | <0.001 |
|  | QRS [ms] | Time (Post vs Pre) | 0.11 | 1.12 | 20.0 | 0.10 | 0.922 |
|  | QRS [ms] | Group (INT vs CON) | 1.28 | 5.33 | 20.8 | 0.24 | 0.812 |
|  | QRS [ms] | Time x Group | -2.20 | 1.52 | 20.0 | -1.45 | 0.162 |
|  | PQ [ms] | Intercept | 183.00 | 8.63 | 22.1 | 21.20 | <0.001 |
|  | PQ [ms] | Time (Post vs Pre) | -0.78 | 3.86 | 20.0 | -0.20 | 0.842 |
|  | PQ [ms] | Group (INT vs CON) | -1.73 | 11.64 | 22.1 | -0.15 | 0.883 |
|  | PQ [ms] | Time x Group | -1.40 | 5.21 | 20.0 | -0.27 | 0.790 |
|  | LVOT [cm] | Intercept | 19.89 | 0.48 | 26.3 | 41.53 | <0.001 |
|  | LVOT [cm] | Time (Post vs Pre) | 0.56 | 0.36 | 20.0 | 1.56 | 0.135 |
|  | LVOT [cm] | Group (INT vs CON) | 0.02 | 0.65 | 26.3 | 0.03 | 0.975 |
|  | LVOT [cm] | Time x Group | -1.10 | 0.48 | 20.0 | -2.29 | 0.033 |
|  | LVIDd [cm/m2] | Intercept | 42.18 | 1.31 | 21.2 | 32.29 | <0.001 |
|  | LVIDd [cm/m2] | Time (Post vs Pre) | 0.22 | 0.45 | 20.0 | 0.49 | 0.626 |
|  | LVIDd [cm/m2] | Group (INT vs CON) | -1.91 | 1.76 | 21.2 | -1.08 | 0.292 |
|  | LVIDd [cm/m2] | Time x Group | 0.32 | 0.61 | 20.0 | 0.53 | 0.599 |
|  | LVM [g] | Intercept | 82.15 | 5.11 | 20.1 | 16.08 | <0.001 |
|  | LVM [g] | Time (Post vs Pre) | 0.18 | 0.50 | 20.0 | 0.36 | 0.724 |
|  | LVM [g] | Group (INT vs CON) | -12.55 | 6.89 | 20.1 | -1.82 | 0.083 |
|  | LVM [g] | Time x Group | 0.40 | 0.67 | 20.0 | 0.60 | 0.557 |
|  | Aorta [mm] | Intercept | 32.56 | 0.80 | 20.4 | 40.74 | <0.001 |
|  | Aorta [mm] | Time (Post vs Pre) | 0.33 | 0.17 | 20.0 | 2.02 | 0.057 |
|  | Aorta [mm] | Group (INT vs CON) | -1.19 | 1.08 | 20.4 | -1.11 | 0.281 |
|  | Aorta [mm] | Time x Group | -0.42 | 0.22 | 20.0 | -1.91 | 0.071 |
|  | IVSd [cm] | Intercept | 9.39 | 0.71 | 21.7 | 13.31 | <0.001 |
|  | IVSd [cm] | Time (Post vs Pre) | 0.57 | 0.28 | 20.0 | 2.02 | 0.057 |
|  | IVSd [cm] | Group (INT vs CON) | 0.03 | 0.95 | 21.7 | 0.03 | 0.976 |
|  | IVSd [cm] | Time x Group | -0.89 | 0.38 | 20.0 | -2.36 | 0.029 |
|  | LA-Volume [ml/m2] | Intercept | 31.15 | 2.12 | 21.3 | 14.70 | <0.001 |
|  | LA-Volume [ml/m2] | Time (Post vs Pre) | 0.11 | 0.76 | 20.0 | 0.15 | 0.886 |
|  | LA-Volume [ml/m2] | Group (INT vs CON) | -1.57 | 2.86 | 21.3 | -0.55 | 0.588 |
|  | LA-Volume [ml/m2] | Time x Group | 0.89 | 1.03 | 20.0 | 0.86 | 0.398 |
|  | Ejection fraction [%] | Intercept | 56.11 | 1.60 | 33.5 | 35.05 | <0.001 |
|  | Ejection fraction [%] | Time (Post vs Pre) | 0.56 | 1.69 | 20.0 | 0.33 | 0.746 |
|  | Ejection fraction [%] | Group (INT vs CON) | 1.43 | 2.16 | 33.5 | 0.66 | 0.511 |
|  | Ejection fraction [%] | Time x Group | 3.99 | 2.29 | 20.0 | 1.75 | 0.096 |
| Lower-limb strength | Leg press 1RM [kg] | Intercept | 85.56 | 10.24 | 22.8 | 8.36 | <0.001 |
|  | Leg press 1RM [kg] | Time (Post vs Pre) | 3.33 | 5.28 | 20.0 | 0.63 | 0.535 |
|  | Leg press 1RM [kg] | Group (INT vs CON) | 7.63 | 13.80 | 22.8 | 0.55 | 0.586 |
|  | Leg press 1RM [kg] | Time x Group | 17.12 | 7.12 | 20.0 | 2.40 | 0.026 |
|  | Leg extension 1RM [kg] | Intercept | 36.11 | 4.69 | 21.6 | 7.69 | <0.001 |
|  | Leg extension 1RM [kg] | Time (Post vs Pre) | -2.22 | 1.83 | 20.0 | -1.21 | 0.239 |
|  | Leg extension 1RM [kg] | Group (INT vs CON) | 0.32 | 6.33 | 21.6 | 0.05 | 0.960 |
|  | Leg extension 1RM [kg] | Time x Group | 9.20 | 2.47 | 20.0 | 3.73 | 0.001 |
| Grip strength and endurance capacity | Handgrip strength [kg] | Intercept | 31.22 | 2.43 | 22.3 | 12.83 | <0.001 |
|  | Handgrip strength [kg] | Time (Post vs Pre) | -2.56 | 1.13 | 20.0 | -2.27 | 0.034 |
|  | Handgrip strength [kg] | Group (INT vs CON) | -5.68 | 3.28 | 22.3 | -1.73 | 0.097 |
|  | Handgrip strength [kg] | Time x Group | 4.56 | 1.52 | 20.0 | 3.00 | 0.007 |
|  | Total time, incremental cycling test [min] | Intercept | 13.67 | 0.93 | 24.9 | 14.66 | <0.001 |
|  | Total time, incremental cycling test [min] | Time (Post vs Pre) | 0.11 | 0.62 | 20.0 | 0.18 | 0.860 |
|  | Total time, incremental cycling test [min] | Group (INT vs CON) | -0.03 | 1.26 | 24.9 | -0.02 | 0.981 |
|  | Total time, incremental cycling test [min] | Time x Group | 2.34 | 0.84 | 20.0 | 2.79 | 0.011 |
|  | Power at 2 mmol/l lactate [W] | Intercept | 36.79 | 9.38 | 19.7 | 3.92 | <0.001 |
|  | Power at 2 mmol/l lactate [W] | Time (Post vs Pre) | 14.36 | 6.14 | 13.6 | 2.34 | 0.035 |
|  | Power at 2 mmol/l lactate [W] | Group (INT vs CON) | 20.09 | 12.67 | 18.9 | 1.59 | 0.129 |
|  | Power at 2 mmol/l lactate [W] | Time x Group | -10.68 | 8.37 | 13.6 | -1.28 | 0.223 |
| Body composition | Body water [kg] | Intercept | 37.44 | 2.18 | 20.5 | 17.16 | <0.001 |
|  | Body water [kg] | Time (Post vs Pre) | 0.84 | 0.50 | 20.0 | 1.68 | 0.109 |
|  | Body water [kg] | Group (INT vs CON) | -0.46 | 2.94 | 20.5 | -0.16 | 0.877 |
|  | Body water [kg] | Time x Group | 0.30 | 0.68 | 20.0 | 0.44 | 0.662 |
|  | Body fat [kg] | Intercept | 24.40 | 2.71 | 20.8 | 9.02 | <0.001 |
|  | Body fat [kg] | Time (Post vs Pre) | -0.70 | 0.76 | 20.0 | -0.92 | 0.370 |
|  | Body fat [kg] | Group (INT vs CON) | 1.68 | 3.65 | 20.8 | 0.46 | 0.650 |
|  | Body fat [kg] | Time x Group | -1.77 | 1.03 | 20.0 | -1.72 | 0.101 |
|  | BCM [kg] | Intercept | 24.78 | 1.85 | 20.1 | 13.41 | <0.001 |
|  | BCM [kg] | Time (Post vs Pre) | -0.18 | 0.22 | 20.0 | -0.82 | 0.425 |
|  | BCM [kg] | Group (INT vs CON) | -0.80 | 2.49 | 20.1 | -0.32 | 0.753 |
|  | BCM [kg] | Time x Group | 1.11 | 0.29 | 20.0 | 3.79 | 0.001 |
|  | CMF [%] | Intercept | 46.21 | 1.42 | 20.2 | 32.57 | <0.001 |
|  | CMF [%] | Time (Post vs Pre) | -0.14 | 0.21 | 20.0 | -0.70 | 0.495 |
|  | CMF [%] | Group (INT vs CON) | 0.25 | 1.91 | 20.2 | 0.13 | 0.896 |
|  | CMF [%] | Time x Group | 0.92 | 0.28 | 20.0 | 3.28 | 0.004 |
|  | Phase angle [°] | Intercept | 4.80 | 0.21 | 21.5 | 23.09 | <0.001 |
|  | Phase angle [°] | Time (Post vs Pre) | -0.04 | 0.08 | 20.0 | -0.55 | 0.586 |
|  | Phase angle [°] | Group (INT vs CON) | 0.02 | 0.28 | 21.5 | 0.06 | 0.949 |
|  | Phase angle [°] | Time x Group | 0.26 | 0.11 | 20.0 | 2.43 | 0.025 |
|  | ECM [kg] | Intercept | 27.58 | 1.09 | 20.4 | 25.22 | <0.001 |
|  | ECM [kg] | Time (Post vs Pre) | -0.01 | 0.22 | 20.0 | -0.05 | 0.959 |
|  | ECM [kg] | Group (INT vs CON) | 0.29 | 1.47 | 20.4 | 0.20 | 0.843 |
|  | ECM [kg] | Time x Group | 0.34 | 0.29 | 20.0 | 1.17 | 0.257 |

Values represent output from linear mixed models with a random intercept per participant and a compound symmetry covariance structure (equal variance at each timepoint, constant covariance between timepoints), fitted separately for each outcome; the Time × Group term represents the primary contrast of interest. The Intercept reflects the model-implied mean for the reference level (CON group, Pre timepoint). 'Time (Post vs Pre)' and 'Group (INT vs CON)' indicate the coding direction of each contrast. SE, standard error; df, degrees of freedom (Satterthwaite approximation); INT, intervention group; CON, control group. 6MWT, Six-Minute Walk Test; TUG, Timed Up and Go; DEMMI, De Morton Mobility Index; PCS, Physical Component Summary; MCS, Mental Component Summary; HR, heart rate; RRsys/RRdia, systolic/diastolic blood pressure; QT/QRS/PQ, electrocardiographic intervals; LVOT, left ventricular outflow tract; LVIDd, left ventricular internal diameter in diastole; LVM, left ventricular mass; IVSd, interventricular septal diameter in diastole; LA, left atrial; 1RM, one-repetition maximum; BCM, body cell mass; ECM, extracellular mass; CMF, cell mass fraction.
