# Supplementary material for: Effects of individualized blood flow restriction during brief twice-weekly low-load cycling on mobility and muscle function in adults older than 70 years
Source: Front Sports Act Living. 2026 Oct 2;8:1933001. doi: 10.3389/fspor.2026.1933001 (PMC13672636; doi:10.3389/fspor.2026.1933001)
Supplement: Supplementary file 3 [file Table3.docx]

**CONSORT 2025 Checklist**

*Effects of individualized blood flow restriction during brief twice-weekly low-load cycling on mobility and muscle function in adults older than 70 years*

| **Section/Topic** | **No** | **CONSORT 2025 checklist item description** | **Reported on page no. / location** |
| --- | --- | --- | --- |
| **Title and abstract** | | | |
| Title and structured abstract | 1a | Identification as a randomised trial | Title |
| Title and structured abstract | 1b | Structured summary of the trial design, methods, results, and conclusions | Abstract |
| **Open science** | | | |
| Trial registration | 2 | Name of trial registry, identifying number (with URL) and date of registration | Abstract; Methods (Trial design); Limitations. Not prospectively registered. Retrospective registration was in progress at the time of this submission; the registration number will be provided prior to publication. Explicitly stated in the Abstract, Methods/Trial design, and Limitations. |
| Protocol and statistical analysis plan | 3 | Where the trial protocol and statistical analysis plan can be accessed | Not reported – no public repository link for the protocol/statistical analysis plan is currently provided. |
| Data sharing | 4 | Where and how the individual de-identified participant data (including data dictionary), statistical code and any other materials can be accessed | Data availability. Individual participant data are available from the corresponding author upon reasonable request, subject to a data-sharing agreement; public deposition is not possible as the informed consent documentation did not cover upload to an external public repository. |
| Funding and conflicts of interest | 5a | Sources of funding and other support (e.g., supply of drugs), and role of funders in the design, conduct, analysis and reporting of the trial | Conflict of Interest; Acknowledgments. No external research funding; article-processing charge covered by the University of Bonn Open Access Publication Fund; BFR device (Personalized Tourniquet System) provided on loan by Delfi Medical Innovations Inc. for the duration of the study, at no cost. The Conflict of Interest statement now explicitly states that the company had no role in study design, conduct, data analysis, or reporting. |
| Funding and conflicts of interest | 5b | Financial and other conflicts of interest of the manuscript authors | Conflict of Interest. No financial or personal relationships that could have inappropriately influenced the work. |
| **Introduction** | | | |
| Background and rationale | 6 | Scientific background and rationale | Introduction |
| Objectives | 7 | Specific objectives related to benefits and harms | Introduction. Aims presented as a numbered list, followed by a single explicit hypothesis statement. |
| **Methods** | | | |
| Patient and public involvement | 8 | Details of patient or public involvement in the design, conduct and reporting of the trial | Not reported – no patient or public involvement in trial design/conduct/reporting is described. |
| Trial design | 9 | Description of trial design including type of trial, allocation ratio, and framework | Methods (Trial design). Prospective, single-blinded, parallel-group RCT, 1:1 allocation. An explicit trial framework (e.g., superiority) is not separately labelled. |
| Changes to trial protocol | 10 | Important changes to the trial after it commenced including any outcomes or analyses that were not prespecified, with reason | Limitations. Not applicable in the conventional sense, as the trial was not prospectively registered (see item 2); the co-primary outcomes (DEMMI, health-related quality of life) and the full analysis plan are stated as having been designated a priori during protocol development, but no external record exists to verify this independently. This is discussed transparently as a limitation. |
| Trial setting | 11 | Settings and locations where the trial was conducted | Author affiliations; Methods (Trial design, Participants). Single-center trial; exercise sessions were conducted in an outpatient setting at the Cellitinnen-Krankenhaus St. Marien, Cologne, Germany (Department of Geriatrics); ethics approval was granted by the Ethics Committee of the German Sport University Cologne. |
| Eligibility criteria | 12a | Eligibility criteria for participants | Methods (Participants). Age >70 years, community-dwelling, written informed consent, medical clearance for BFR exercise; exclusion criteria include malignancy, immunosuppression, systemic infection, sickle cell anemia, and prior lower-extremity vascular surgery. |
| Eligibility criteria | 12b | Eligibility criteria for sites and for individuals delivering the interventions | Methods (Interventions). Sessions supervised by a sports therapist; all outcome assessments performed by a single sports therapist, blinded to group allocation, who completed standardized official DEMMI rater training and was not involved in training supervision. Formal eligibility criteria for delivering personnel (e.g., certification requirements) are not separately specified. |
| Intervention and comparator | 13 | Intervention and comparator with sufficient details to allow replication | Methods (Interventions; Limb Occlusion Pressure and Blood-Flow-Restriction). Individualized cuff pressure at 60% of measured limb occlusion pressure (INT) vs. fixed 20 mmHg sham pressure (CON), delivered via an autoregulating tourniquet system (Delfi Medical Innovations), on a stationary cycle ergometer; internal-load-matched intensity prescription described in detail, including the session-to-session workload adjustment algorithm. |
| Outcomes | 14 | Pre-specified primary and secondary outcomes, including measurement variable, analysis metric, aggregation, and time point | Methods (Outcomes; Outcome measures). Co-primary outcomes are functional mobility (DEMMI) and health-related quality of life (SF-36, all eight domains/summary scores analyzed jointly); secondary outcomes are TUG, 6MWT, muscular strength (1RM leg press/leg extension, handgrip), body composition (BIA), endurance capacity, and cardiovascular/metabolic safety parameters. Outcomes are also fully tabulated with instrument, original developer reference, unit, and reliability evidence in Supplementary Table S1. |
| Harms | 15 | How harms were defined and assessed | Methods (Cardiovascular safety); Limitations. Adverse events, dropouts, and resting cardiovascular/ECG/echocardiographic findings assessed systematically pre- and post-intervention; acute cardiovascular responses during BFR-cycling sessions themselves were not systematically monitored, which is explicitly stated as a limitation on the scope of the safety conclusions. |
| Sample size | 16a | How sample size was determined, including all assumptions | Methods (Sample Size Calculation). G*Power-based calculation using published effect sizes for BFR-induced muscle strength gains (the co-primary outcomes could not be directly powered due to the absence of pilot data; this is stated explicitly, together with the resulting precision limitation). |
| Sample size | 16b | Explanation of any interim analyses and stopping guidelines | Not applicable – no interim analyses or stopping guidelines were used. |
| **Randomisation** | | | |
| Sequence generation | 17a | Who generated the random allocation sequence and the method used | Methods (Participants, Randomization). Computer-generated randomization sequence (www.randomizer.org). |
| Sequence generation | 17b | Type of randomisation and details of any restriction | Methods (Participants, Randomization). 1:1 allocation stated; block size/stratification not separately reported. |
| Allocation concealment mechanism | 18 | Mechanism used to implement the random allocation sequence | Methods (Participants, Randomization). Sequentially numbered, opaque, sealed envelopes. |
| Implementation | 19 | Whether personnel who enrolled/assigned participants had access to the allocation sequence | Methods (Participants, Randomization). Envelopes prepared by an investigator not involved in recruitment or testing; who specifically enrolled and assigned participants is not further detailed. |
| Blinding | 20a | Who was blinded after assignment to interventions | Methods (Trial design; Outcomes). A single outcome assessor performed all functional, mobility, strength, and exercise testing (DEMMI, TUG, 6MWT, 1RM, handgrip, endurance test) and was blinded to group allocation throughout; a separate single investigator analyzed echocardiographic parameters, also blinded. Participants and the supervising therapist were not blinded. Participant blinding could not be fully guaranteed given the perceptible difference in cuff pressure (INT 121 ± 14 mmHg vs. CON 20 mmHg); no formal post-intervention blinding check was performed (explicitly noted as a limitation). |
| Blinding | 20b | If blinded, how blinding was achieved and description of the similarity of interventions | Methods (Trial design; Outcomes). Identical exercise protocol and identical tourniquet device/procedure in both groups; CON received the same cuffs at a fixed, non-occlusive sham pressure of 20 mmHg rather than no cuff; participants could not view the device display during inflation and were informed only that the study compared a higher- and a lower-pressure condition. |
| **Statistical methods** | | | |
| Statistical methods | 21a | Statistical methods used to compare groups for primary and secondary outcomes, including harms | Methods (Statistics). Linear mixed models (time × group interaction) as the primary analysis, with baseline-adjusted ANCOVA as a pre-specified sensitivity analysis; Benjamini-Hochberg correction applied within outcome families (SF-36, laboratory, ECG, echocardiography, body composition, strength/endurance); the DEMMI (single composite score) and the individually-tested TUG/6MWT are tested at the nominal alpha level. Both standardized (Hedges' g) and unstandardized (adjusted mean difference) effect sizes with 95% CIs are reported for all contrasts, with an explicit explanation of why the two interval types can differ in precision. |
| Statistical methods | 21b | Definition of who is included in each analysis, and in which group | Results. All 20 randomized participants (11 INT / 9 CON) completed the intervention and were included in the analysis on an intention-to-treat basis; no exclusions after randomization. |
| Statistical methods | 21c | How missing data were handled in the analysis | Methods (Statistics). Missing data were handled via maximum likelihood estimation within the linear mixed models (missing-at-random assumption), without imputation. Missing data occurred for the workload at 2 mmol·L⁻¹ blood lactate (13 of 20 participants with complete pre-post pairs; 7 excluded due to elevated baseline lactate) and for SF-36 (1 participant did not complete the questionnaire); per-group denominators are reported wherever this outcome appears. |
| Statistical methods | 21d | Methods for any additional analyses, distinguishing prespecified from post-hoc | Methods (Statistics). Baseline-adjusted ANCOVA reported as a pre-specified sensitivity analysis for all primary and secondary outcomes; no additional subgroup analyses were performed. |
| **Results** | | | |
| Participant flow, including flow diagram | 22a | For each group, the numbers randomly assigned, receiving intended intervention, and analysed | Results; Figure 1 (CONSORT flow diagram). 27 assessed for eligibility, 7 excluded, 20 randomized (11 INT / 9 CON), all analyzed. |
| Participant flow, including flow diagram | 22b | For each group, losses and exclusions after randomisation, with reasons | Results; Figure 1 (CONSORT flow diagram). No losses, dropouts, or exclusions after randomization; all 20 randomized participants completed the six-week intervention. |
| Recruitment | 23a | Dates defining the periods of recruitment and follow-up | Not reported – specific recruitment start/end dates are not stated in the manuscript. |
| Recruitment | 23b | If relevant, why the trial ended or was stopped | Not applicable – the trial was completed as planned. |
| Intervention and comparator delivery | 24a | Intervention and comparator as actually administered, including fidelity | Methods (Interventions); Discussion (Cardiovascular safety). Twelve supervised 15-minute sessions completed by all participants (100% session completion, no dropouts); the session-to-session workload adjustment protocol (internal-load matching) is described in detail. Formal per-session fidelity/adherence logs beyond total session completion are not reported. |
| Intervention and comparator delivery | 24b | Concomitant care received during the trial for each group | Limitations. Not systematically documented. Heart-rate-limiting medication (e.g., beta-blockers, other antihypertensives), which is directly relevant given the heart-rate-based intensity prescription, was not recorded and could not be accounted for; this is stated explicitly as a limitation. |
| Baseline data | 25 | A table showing baseline demographic and clinical characteristics for each group | Table 1. Groups were well balanced at baseline (p ≥ 0.05 for all comparisons) with the exception of the SF-36 Vitality domain (p = 0.012) and a numerically large, though non-significant, difference in handgrip strength (p = 0.091); both are discussed explicitly in Results, Discussion, and Limitations. |
| Numbers analysed, outcomes and estimation | 26 | For each outcome, by group: number analysed, number with available data, result, and estimated effect size with precision | Results; Tables 2–5. For every outcome, group means/SDs at each timepoint, main/interaction effect p-values (with Benjamini-Hochberg q-values where applicable), and both standardized (Hedges' g) and unstandardized (adjusted mean difference) effect sizes with 95% CIs are reported; per-group denominators are given, including for outcomes with missing data (e.g., W/2mmol). |
| Harms | 27 | All harms or unintended events in each group | Discussion (Cardiovascular safety); Conclusions. No dropouts, adverse events, or clinically relevant adverse findings on resting cardiovascular/metabolic monitoring in either group. |
| Ancillary analyses | 28 | Any other analyses performed, including subgroup and sensitivity analyses | Methods (Statistics). Baseline-adjusted ANCOVA reported throughout as a pre-specified sensitivity analysis for all outcomes; no additional post-hoc subgroup analyses were performed. |
| **Discussion** | | | |
| Interpretation | 29 | Interpretation consistent with results, balancing benefits and harms, considering other evidence | Discussion; Conclusions. Interpretation is calibrated to effect precision throughout (e.g., the DEMMI result is described as closely approaching, rather than exceeding, the minimal clinically important difference; the perceived-exertion finding is explicitly framed as hypothesis-generating rather than confirmatory); a dedicated paragraph addresses all non-significant ("null") between-group comparisons as findings in their own right. |
| Limitations | 30 | Trial limitations, addressing bias, imprecision, generalisability, and multiplicity | Discussion (Limitations). Presented as a numbered list of ten points, covering sample size/power, baseline imbalance, absence of prospective registration, blinding and rater reliability, multiplicity across outcome families, body-composition methodology, heart-rate-formula error and unrecorded medication, echocardiographic reader reliability, endurance-test stopping-criterion documentation, and absence of follow-up; interpretation of the SF-36 across cohorts/eras is additionally addressed as a distinct measurement-validity limitation. |
